# Offline text-independent writer identification using a codebook with structural features

**DOI:** 10.1371/journal.pone.0284680

**Published:** 2023-04-25

**Authors:** Bashar Q. Ahmed, Yaser F. Hassan, Ashraf S. Elsayed

**Affiliations:** 1 Department of Mathematics and Computer Science, Faculty of Science, Alexandria University, Alexandria, Egypt; 2 Faculty of Computing and Data Science, Alexandria University, Alexandria, Egypt; 3 Faculty of Computer Science and Engineering, Al Alamein International University, Marsa Matroh, El Alamein, Egypt; Military Institute of Science and Technology, BANGLADESH

## Abstract

Understanding handwritten documents is a vital and challenging problem that attracts many researchers in the fields of forensic and authentication science. This paper presents an offline system for text-independent writer identification of handwritten documents. The system extracts a handwritten connected component contour, which in turn is divided into segments of specific length. The system utilizes the concept of a bag of features in the writer recognition domain and considers handwritten contour segments to extract two conceptually simple and effective structural features. These features are the contour point curve angle and the CONtour point CONcavity/CONvexity. The system uses the proposed features to train a k-means clustering algorithm to construct a codebook of size *K*. The method then uses occurrence histograms of the extracted features in the codebook to create a final feature vector for each handwritten document. The effectiveness of the proposed features is evaluated in the writer identification domain using two widely used classification methods: the nearest neighbor and the support vector machine techniques. The proposed writer identification is evaluated on two large and public datasets from different language domains, the Arabic KHATT and English IAM datasets. The experimental results show that the proposed system outperforms state-of-the-art methods on the IAM dataset and provides competitive results on the KHATT dataset with respect to the identification rate.

## 1 Introduction

Manual handwriting analysis depends mainly upon the identification of distinguishing patterns in a handwriting sample, which in turn can be compared with the patterns encapsulated in a new handwritten document from a known writer [1]. The rapid developments in computing resources enable forensic society to develop automated systems to replace manual handwriting analysis. Hence, many systems have been developed to solve many problems connected to forensic science. Some of these systems deal directly with the problem of writer identification as a part of forensic science, while the remaining deal with other fields.

Text recognition is also a wide problem in which extensive studies have been done, and some of these studies address the writer recognition topic in one way or another. Writer recognition involves many problems, such as writer identification, writer verification, writer retrieval, handedness classification, and gender classification [2]. Writer identification is the task of assigning an unknown handwritten document to a specific scribe from a group of known scribes. Writer verification is the task of comparing a sample handwritten document with another handwritten document from a known author for the sake of accepting or rejecting the authority of its scribe [3]. According to this definition, the writer identification problem is a one-to-many classification problem, while writer verification is a one-to-one classification problem, which implies that the identification problem is harder than the verification problem, as reported in [4, 5].

Over time, a writer pursues a specific writing style that encapsulates some specific features characterizing his or her writing style. An example of these features is the handwritten text curvature and the appearance frequency of some basic details, such as letters or even parts of letters [6]. Hence, writer identification can be used as a biometric technique, as the writer usually follows some writing styles that differ from other styles adopted by other writers [3].

Writer identification gains importance from a variety of applications. Such applications include forensic analysis [7], historical document analysis [8], musical score analysis [9], and personalized recognition systems [10], and this importance encourages the research community to pay intensive attention to writer identification. As a result, a plethora of different valuable approaches has been proposed. However, writer identification is still a hard and challenging problem due to two main facts. First, some languages have different writing styles, such as the Arabic language, which can be written in several styles, including NASKH, REQA’A, and FARISI. Furthermore, some languages can be written in cursive or separated letters manner. Second, each writer is subjected to different writing qualities or interwriter variations over a short-term period. Moreover, the writing habit adopted by the same writer can be developed and changed over a long-term period [11, 12].

In this paper, an effort has been made to develop an automatic system for offline text-independent writer identification for Arabic and English handwritten scripts using the concept of a codebook with structural features. To perform this objective, an insightful literature review of existing methods was performed to identify and solve the lacking aspects from the domain. We found that although there are many studies on Arabic handwritten writer identification, very few studies have been performed on a large Arabic dataset that consists of variants of information. Furthermore, there is an observation in some state-of-the-art studies about the superiority of considering the analysis of small fragments of handwriting images over the analysis of the global texture of handwriting images. Therefore, we designed two effective features to capture some properties of small segments from the connected component contour of handwritten documents to check and confirm the state-of-the-art observation. In summary, our contributions can be listed as follows:

- A comprehensive literature review was performed to identify the lacking aspects in the writer identification field. This review provides insightful comments on related studies, highlighting the major contributions and limitations.
- Two effective structural features are proposed. The first feature is called the CONtour point CONcavity/CONvexity (CON^3^), which is extracted based on the concavity and convexity of a handwriting contour. The second feature represents the contour point curve angle (CPCA).
- A system to utilize the proposed features in the writer identification domain is designed.
- The proposed writer identification method is applied to two large datasets, the English IAM and the Arabic KHATT, which is a large dataset that consists of variants of information.

The paper is organized as follows. The literature contributions to writer identification are introduced in section 2. Section 3 presents the proposed method in detail. The datasets and the experimental results are discussed in sections 4 and 5, respectively. Finally, the paper is concluded in section 6.

## 2 Related work

As stated earlier, the importance of the writer identification problem encourages the research community to pay close attention to it. Many studies have been proposed during the last few decades. Different aspects can be used to categorize these studies and to identify their pros and cons.

According to the type of handwritten document sample, writer identification systems can be broadly categorized into online and offline writer identification. Online writer identification is performed during the writing process using special devices such as tablets. On the other hand, offline writer identification uses digitized images of handwritten documents after the writing process itself. Offline identification is harder than online identification due to the lack of some information that is available for online identification, such as velocity, pen pressure, writing trajectory, etc. [13]. Using textual content, writer identification can be divided into text-dependent and text-independent techniques [11]. In text-dependent writer identification, writers have to copy the same text contents that are then used in training and testing. On the other hand, text-independent writer identification imposes no text content restrictions. Therefore, text-independent writer identification is the closest to real-world scenarios, as reported in [14].

Considering the type of features as a functional context, the methods reported in the literature can be categorized into four main groups: structural-based, textural-based, grapheme-based, and auto-learned based methods. Structural-based methods consider the allograph shapes and then apply a grapheme-emission probability distribution. For this reason, some studies in the literature referred to these methods as statistical methods, which usually require some preprocessing steps to extract the structural features. Structural-based systems have been noted to achieve better identification rates, although this occurs at the cost of run time due to the complexity of the preprocessing steps [11]. Such preprocessing steps include binarization, edge detection, and segmentation. On the other hand, textural-based systems are more efficient regarding the runtime, as they do not need additional steps such as segmentation but are known to have lower identification rates [15].

Structural-based methods can be further categorized into connected component-based methods [16–19], contour direction-based methods [16, 20], and contour pattern-based methods [19, 21]. Although structural-based methods are more intuitive and stronger, these methods are very vulnerable to any slight variations in the character or allograph characteristics, such as slant or aspect ratio. Additionally, these methods concentrate mainly on the properties of the allographs or characters themselves, which means neglecting important information represented by the properties between allographs drawn in the same word. Furthermore, relying on preprocessing steps is considered a major limitation of these methods, since if the preprocessing step fails, then by necessity, all successive steps, such as feature extraction and classification, are subject to failure.

He et al. [22] proposed a novel feature in which they specified a reference point inside the handwritten text and then calculated the stroke-length distribution in each direction surrounding that reference point. Their proposed feature (Junclet) is said to be a simple and efficient local descriptor. It is worth mentioning that their method can be considered one of the few methods that touch on cross-script writer identification. The method is evaluated on a new challenging dataset in which each writer participates in writing text using English and Chinese languages. The authors reported that the Junclet feature is scale- and rotation-invariant, and the experiments proved that the Junclet’s atomic elements are promising in different applications related to text recognition. Khalifa et al. [13] presented a new method for writer identification that employs multiple codebooks instead of a single codebook. The algorithm uses kernel discriminant analysis using spectral regression SR-KDA for the sake of reducing features vector dimensionality to avoid the overfitting problem. The method was evaluated on two public datasets: IAM and ICFHR. The experiments proved that the fusion of multiple codebooks significantly enhances the performance rate. During the experiments, they noted that fusing multiple codebooks achieves better performance than a single codebook by almost 11%. Graz et al. [23] presented a simple interest point-based system for writer identification using novel descriptors that capture the geometric relationships among different parts of handwritten documents. The descriptors exactly represent the probability density functions of the distribution of strokes, junctions, endings, and loops. The proposed system has the advantages of simplicity and efficiency but at the cost of the need for a large amount of data to acquire stable models. The system was evaluated on four datasets: IAM, ICDAR’11F, ICDAR’11C, and ICDAR’13, and the authors reported that the results are comparable to the results achieved by a complex interest point-based system from the literature. Tang et al. [24] presented a system for writer identification based on some structural features. The system extracts the SIFT features and then modifies the SIFT descriptors by replacing the dominant orientation with the real orientation of an interest point. The TD descriptor was proposed to capture the relationships among the contour points of three words. The TD descriptor is a modification of the well-known hinge feature proposed in [25]. The proposed system was evaluated on two public datasets from different language domains, and the experimental results show that the proposed method is comparable to the state-of-the-art methods on the same datasets. Djeddi et al. [26] proposed a system that uses edge hinges, edge directions, and run-length features. The extracted features are then used by the multiclass support vector machine (SVM) classifier, and the proposed system is one of the first systems to be applied on a large Arabic dataset (KHATT) involving handwritten documents from 1000 participants. The experiment reported that the combination of run-length and edge-hinge features achieved the best results at 84.10%.

Textural-based methods are sometimes referred to as transformation methods [27] because handwritten script images are considered special textures, and some transformation techniques are applied before feature extraction. Textural-based methods are more efficient concerning the runtime, as they do not need additional preprocessing steps, such as segmentation, but are noted to have lower identification performance [15]. Furthermore, textural-based methods usually need more data to extract reliable and highly discriminative features, which is not the situation in real-world scenarios where forensic experts often encounter small pieces of text to be examined against the writer’s identity.

Hannad et al. [11] presented a textural-based system for writer identification from handwritten documents. The system first divides a handwritten document into small fragments and then individually considers each small fragment as a texture. The proposed system is evaluated against the Arabic IFN/ENIT [28] and English IAM [29] datasets. The authors reported that the proposed system achieves 94.89% on IFN/ENIT and 89.54% on IAM using the complete set of writers from the two datasets. Chahi et al. [30] proposed an offline handwriting writer identification system in which the learning method exploits small local regions of the handwritten document. The document is segmented into connected components, which in turn are fed into a cross multiscale locally encoded gradient patterns (CLGP) operator to compute a feature vector. As a classifier, they applied a dissimilarity measure using the Hamming distance, and the system was evaluated on six public datasets from different domains. The proposed descriptor achieved the highest results on some datasets and achieved competitive results on other datasets. Abbas et al. [31] proposed a system for multiscript text-independent offline writer identification. The proposed system builds a column histogram by crossing local binary patterns (LBP) with different parameters settings to capture the local textural information from a handwritten sample. This operator is then augmented with the oriented basic image features (oBIFs) column histogram. For the classification task, the system employs a multiclass SVM and realizes results competitive with state-of-the-art methods. Christlein et al. [14] proposed a system for offline writer identification using a local descriptor called RootSIFT to capture the local properties of an individual’s handwritten documents. The system replaced the kernel feature of the GMM model with SIFT descriptors and then generated GMM supervectors for each handwritten document. The proposed algorithm employs the Exemplar-SVM to train and test document-specific similarity measures. The system was evaluated on three datasets, ICDAR, CVL, and KHATT, and the performance was shown to surpass existing methods that were evaluated on the same datasets. Tang et al. [24] proposed a textural-based system using two textural descriptors: the stroke fragment histogram (SFH) and local contour pattern histogram (LCPH). The former descriptor is calculated based on a codebook, while the latter descriptor is calculated from the image’s contour points. As a result, the system achieved identification rates equal to 91.3% and 85.4% using the SFH and LCPH descriptors, respectively.

Grapheme-based methods, sometimes referred to as bags of features BOF-based methods, depend mainly on codebook generation for a bag of words (BOW). Methods [17, 25, 32] are examples of this type of writer identification. In this type of method, the handwritten text of the script is first segmented into small segments (graphemes) of text, and a codebook is generated using any clustering algorithm. Each segment from the underlying script is assigned to exactly one codeword from the codebook, and the resulting codeword histogram of the script is finally used to predict the script identity. Grapheme-based methods usually involve the computation of a local descriptor via grapheme blobs, vector quantization using clustering, script representation using histograms of vectors, dissimilarity measurements, and classifier learning. However, this type of writer identification method suffers from some drawbacks represented by the need to spend much time extracting and comparing grapheme details. Additionally, due to the large number of grapheme features, this type of method requires substantial memory, especially for methods that apply a single clustering algorithm [13].

He & Schomaker [6] were inspired by the fact that joining features distribution of two or more properties can lead to better performance of the system. Accordingly, they proposed a method using two novel curvature-free features: the run-length of local binary patterns (LBPruns) and the cloud of line distribution (COLD). The former captures the joint distribution of the traditional run-length and local binary patterns, while the latter captures the joint distribution of the relations between the length of the line segment and its orientation. The system was evaluated on three datasets, CERUG, IAM, and Firemaker. Bennour et al. [33] proposed a system for offline text-dependent and text-independent writer identification using the concept of an implicit shape codebook. The proposed system applies the Harries detection technique to specify the most dominant points of the input handwritten image. Patches or windows around the detected points are specified and clustered to build an implicit shape codebook. The system was evaluated on the BFL and CVL datasets and promising results were reported.

Auto learned methods, sometimes called model-based methods rely on the usage of deep learning techniques to extract automatic features learned by deep models. The main drawback of deep models is the need for enormous and massive labeled data to learn, which is not the case in all benchmarks that are usually used in the writer identification task [30]. According to [34], another important drawback of deep techniques is the difficulty associated with selecting the best values for a large number of parameters. Additionally, this type of techniques requires very high computational time compared to the traditional handcrafted methods [35, 36]. Furthermore, methods that apply the auto learned techniques did not win the competitions organized by the 2016 International Conference on Pattern Recognition (ICPR2016) and the 2017 International Conference on Document Analysis and Recognition (ICDAR2017). For these reasons, traditional learning methods perform better than deep ones with respect to the writer identification task as reported in [35].

The literature on the writer identification field is not rich with studies that use such deep learning techniques. However, some important studies were designed based on deep techniques. The methods presented in [37–40] were the first methods that introduce deep learning in the writer identification task. All these methods applied the activation layer of a trained convolutional neural network as features. At that time, these recent studies that apply the auto learned techniques did not set a new performance standard with respect to the identification rate on the well-known benchmarks or in the competitions that were organized such as ICDAR2011, ICDAR2013. However, as new techniques, they show promising and comparative results to that achieved by other methods using handcrafted features. In [38], the CNN features are computed from handwritten image patches, and then, they apply GMM encoding to generate the features vectors as an input to the classification step. In another work [39], the authors apply the SIFT technique to specify the key points and then, they introduce sub-image patches around the key points to a deep CNN for the sake of extracting auto-learned features from the activations of the hidden layers.

## 3 Proposed method

Inspired by the high identification rate achieved by structural-based methods and the discrimination power of considering small writing ink strokes in characterizing writers [8, 11, 41], this paper presents a method for offline text-independent writer identification using Arabic and English handwriting samples. This technique is mainly based on local analysis of handwriting ink strokes using two features, the contour points curve angle (CPCA) and CONtour point CONcavity/CONvexity (CON^3^).

**Fig 1:**
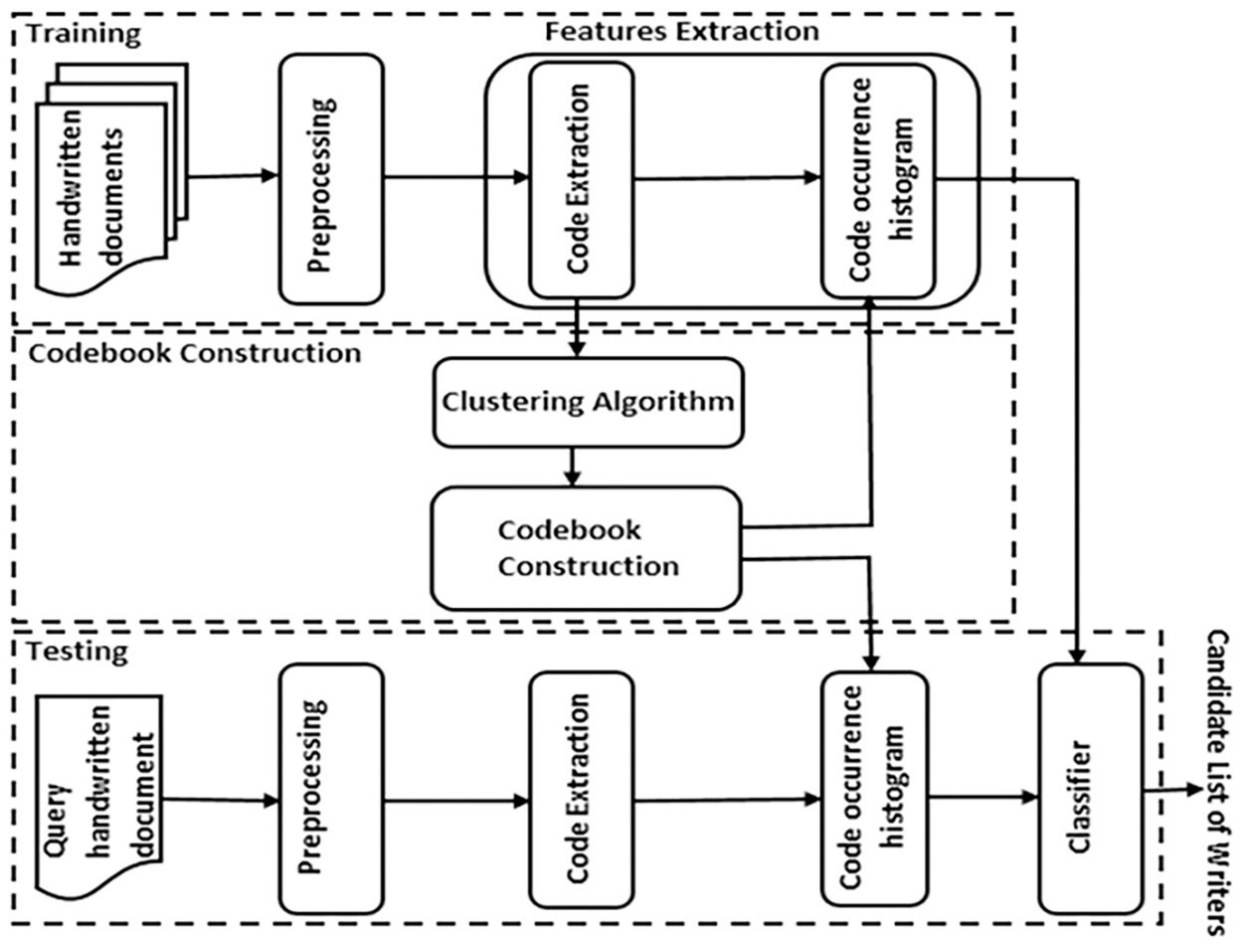
Overview of the proposed method.

As shown in Fig 1, the proposed writer identification method mainly involves three phases: training, codebook generation and testing. For the training phase, the proposed method applies preprocessing and structural features extraction. Additionally, the training phase involves the calculation of the occurrence histogram in which the most similar codeword in the codebook is specified and the corresponding occurrence histogram bin is incremented. Having extracted all features from the training dataset, the proposed method generates a codebook using a simple clustering technique called k-means clustering to cluster features into K clusters. All clusters’ centers compose the codebook, and each cluster’s center forms a single codeword. Similarly, an evaluation handwritten document in the testing set is first preprocessed, and its structural features are extracted to calculate their occurrence histogram. Having both training and testing occurrence histograms, the trained classifier can then retrieve a candidate list of the possible writers.

As preprocessing steps, this method applies the average filter on the scanned images, and then the images are binarized using Otsu thresholding [42], which is an efficient parameterless global binarization method. The Otsu technique is one of the most popular techniques that are used with clean modern handwriting images. Schomaker et al. [25] found that there are no significant differences in binarized images obtained using Otsu, AdOtsu [42, 43] and other binarization methods [44, 45] on modern handwritten document images. Therefore, this paper adopts the Otsu threshold method for image binarization, and the connected components are detected using the 8-neighborhood connectivity strategy. The next subsections explain in detail the most dominant components of the proposed method.

### 3.1 Features extraction

Two features are extracted from ink stroke segments while traveling on the connected component contour. Each feature exploits a different curve’s attributes to characterize the handwriting style.

#### 3.1.1 CPCA Feature extraction

**Fig 2:**
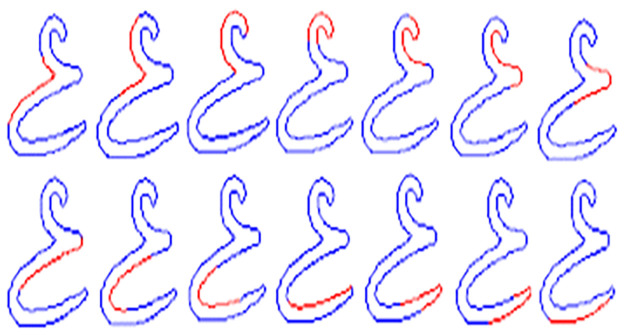
Contour curve fragments of the arabic letter ain.

**Fig 3:**
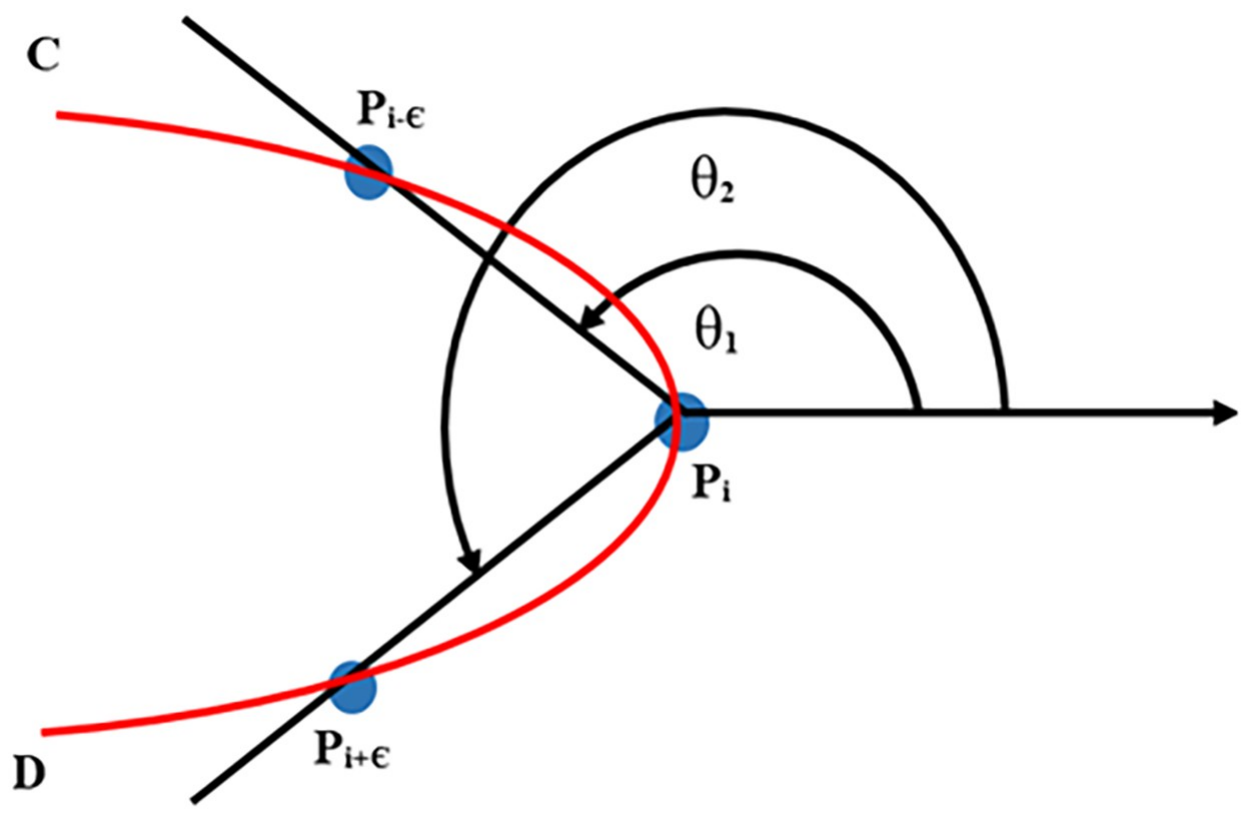
How to compute CPCA feature attributes at point *P*_*i*_.

To extract this feature, the proposed method splits the connected component contour into small fragments of a specific length equal to the value specified by the *NP* parameter. The segmentation process starts at the contour’s first point and uses the *NP* parameter to specify the fragment size. The next segmentation start point is specified with the *GAP* parameter, which specifies the distance between the starting points of each two successive contour fragments, as depicted in Fig 2. It is worth mentioning that the number of features extracted from the connected component contour can be controlled by the *NP* and *GAP* parameters. To calculate the feature vector, this method considers the extracted contour fragment’s points and then calculates the curve angle at each point *P*_*i*_ of the extracted fragment. To do so, this method uses two line fragments of length *Є*. One of them represents an inbound line fragment *P*_*i*−Є_*P*_*i*_ to the point of interest *P*_*i*_, while the other represents an outbound line fragment *P*_*i*_*P*_*i*−Є_ from *P*_*i*_, as illustrated in Fig 3.

As shown in Fig 3, the inbound line fragment P_i−Є_P_i_ creates an angle *θ*_*1*_ with the horizontal axis, and the outbound line fragment P_i_P_i+Є_ creates an angle *θ*_*2*_ with the horizontal axis. *θ*_*1*_ and *θ*_*2*_ can be calculated using (1) and (2), respectively.


$$\theta_{1}=arctan\left(\frac{y_{i}-y_{i-Є}}{x_{i}-x_{i-Є}}\right)$$ (1)

$$\theta_{2}=arctan\left(\frac{y_{i+Є}-y_{i}}{x_{i+Є}-x_{i}}\right)$$ (2)

As illustrated in Fig 3, using *θ*_*1*_ and *θ*_*2*_, the curve angle at point *P*_*i*_ can be estimated using (3).


$${\text{Ф}}_{2\pi }\left(P_{i}\right)=min\left(abs\left(\theta_{2}-\theta_{1}\right),\;2\pi -abs\left(\theta_{2}-\theta_{1}\right)\right)$$ (3)

This procedure is performed for all points of each contour fragment. Vectors containing the curve angles of each contour fragment have the dimensionality of the *NP* parameter. We think that using the resulting curve angle vector as a feature directly is not useful. The main reason is that the comparison of curve angle features in a point-to-point manner is very sensitive to variability among different handwritten documents of the same writer. Therefore, the resulting curve angle vector is quantized into a number of angular intervals *M*. This vector is then augmented with both *θ*_*1*_ and *θ*_*2*_ angles, which in turn are quantized into *N*_*a*_ angular intervals. Accordingly, the final CPCA vectors for all contour fragments are all of dimensionality *M+2N*_*a*_ and are used as features of the handwritten document image.

#### 3.1.2 CON^3^ Feature extraction

**Fig 4:**
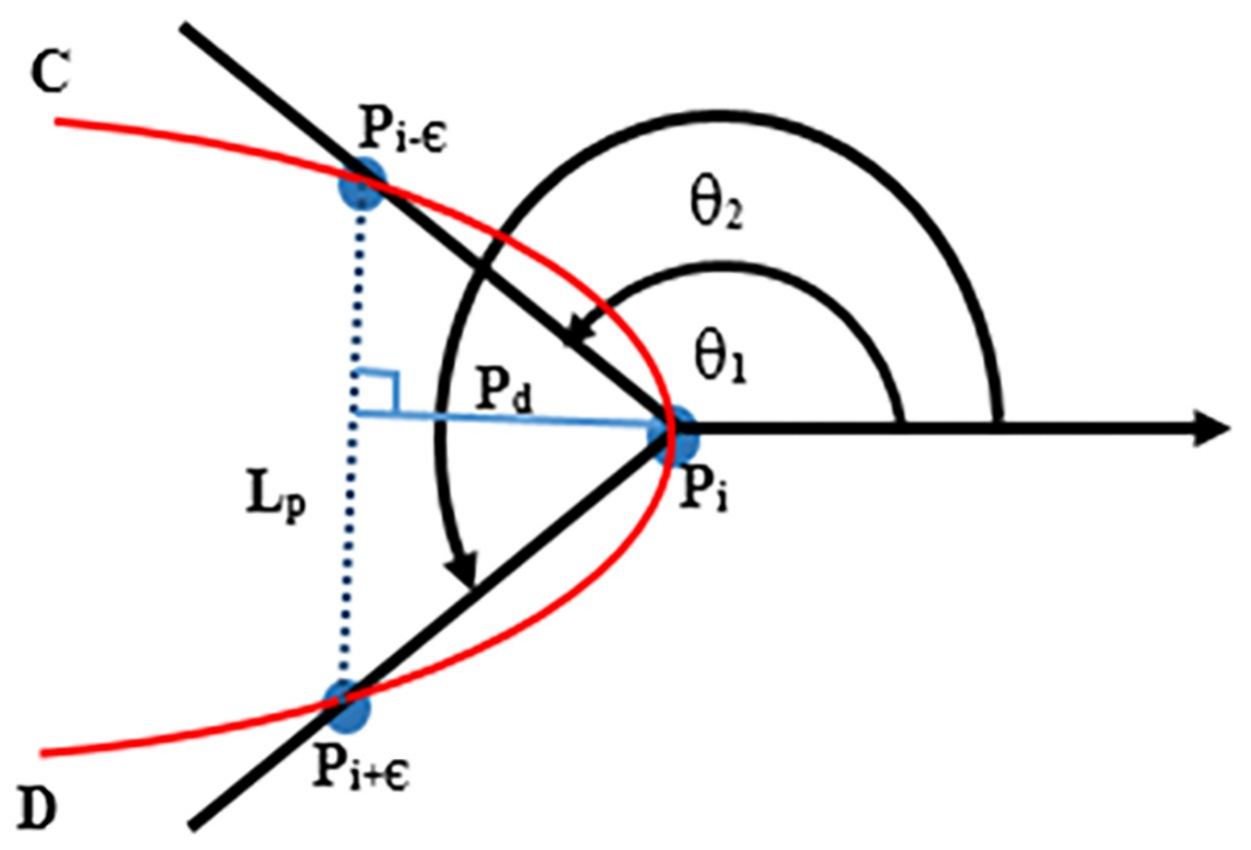
How to compute CON^3^ feature attributes at point *P*_*i*_.

Similar to CPCA feature extraction, this method splits a connected component contour into small fragments of a specific length equal to the value specified by the *NP* parameter. Additionally, the contour is segmented into small fragments using the same parameters referred to in the CPCA feature extraction. To calculate a feature vector, the method considers the points of a contour fragment and then calculates the concavity/convexity property at each point *P*_*i*_ of the contour’s fragment. For this purpose, the method two line fragments of length Є. One of them represents an inbound line fragment *P*_*i*−Є_*P*_*i*_ to the point of interest *P*_*i*_, while the other represents an outbound line fragment *P*_*i*_*P*_*i*+Є_ from P_i_, as illustrated in Fig 4. To measure the concavity/convexity property at point *P*_*i*_, this method measures the perpendicular distance from point *P*_*i*_ to the straight line that connects both *P*_*i*−Є_ and *P*_*i*+Є_ points.

According to [46], this perpendicular distance *P*_*d*_ is given by (4) on the basis of *P*_*i*_ *= (x*_*i*_, *y*_*i*_*)*, *P*_*i-Є*_ *= (x*_*i-Є*_, *y*_*i-Є*_*)* and *P*_*i+Є*_ *= (x*_*i+Є*_, *y*_*i+Є*_*)*.


$$P_{d}=\frac{abs\left(\left(x_{i+Є}-x_{i}\right)\left(y_{i}-y_{i-Є}\right)-\left(x_{i}-x_{i-Є}\right)\left(y_{i+Є}-y_{i}\right)\right)}{sqrt\left({\left(x_{i+Є}-x_{i}\right)}^{2}+{\left(y_{i+Є}-y_{i}\right)}^{2}\right)}$$ (4)

In addition to the perpendicular distance *P*_*d*_, this method also uses the length of line *L*_*p*_ connecting both *P*_*i-Є*_ and *P*_*i+Є*_ points as one attribute of the final feature vector. The line *L*_*p*_ length is given by (5) on the basis of *P*_*i-Є*_ *= (x*_*i-Є*_, *y*_*i-Є*_*)* and *P*_*i+Є*_ *= (x*_*i+Є*_, *y*_*i+Є*_*)* [6].


$$L_{p}=sqrt\left({\left(y_{i+Є}-y_{i-Є}\right)}^{2}+{\left(x_{i+Є}-x_{i-Є}\right)}^{2}\right)$$ (5)

Furthermore, this method augments the final feature vector with the angles *θ*_*1*_ and *θ*_*2*_ formed by the line fragments *P*_*i-Є*_*P*_*i*_, *P*_*i*_*P*_*i+Є*_ and the horizontal axis. Angles *θ*_*1*_ and *θ*_*2*_ can be calculated using (1) and (2), respectively. This procedure is performed for all points of each contour fragment. Vectors containing the perpendicular distance *P*_*d*_, line *L*_*p*_, *θ*_*1*_ and *θ*_*2*_ of each contour fragment have dimensionality *N*_*P*_ multiplied by 4. To reduce the dimensionality of the final vector and reduce the sensitivity to variability among the handwritten samples of the same writer, this method applies the concept of quantization on each of the four attributes separately. Perpendicular distance *P*_*d*_ and line *L*_*p*_ are quantized into *N*_*q*_ and *N*_*l*_ distance intervals, respectively, and *θ*_*1*_ and *θ*_*2*_ are quantized into *N*_*a*_ angular intervals. Therefore, the final CON^3^ vectors are all of dimensionality *N*_*q*_*+N*_*l*_ *+2N*_*a*_ and are used as features for the handwritten document image.

### 3.2 Codebook generation and features vector computation

This method constructs a codebook using the extracted features from the entire training set. The extracted features are used to train a clustering algorithm, and a specific number *K* of clusters is determined. According to [47], many clustering algorithms have been investigated and no significant performance differences were noted among the investigated algorithms. Hence, this method uses the k-means clustering algorithm for clustering purposes due to its simplicity, and popularity in the field, and the resultant *K* clusters then represent the size of the constructed codebook.

To calculate the final feature vector of the handwritten document, we only need to construct an occurrence histogram with a number of bins equal to the *K* codewords. To do so, the features extraction is first applied to the handwritten document, and then, the similarity between each extracted feature and each codebook codeword is determined using the Euclidean distance measurement. Then, the most similar codeword is specified, and its corresponding bin in the histogram is incremented by one. Finally, the constructed histogram represents the final feature vector of the handwritten document with dimensionality equal to the size of the codebook.

## 4 Datasets

The experimental study of the proposed method is carried out on two public datasets: KHATT [48] from an Arabic background and IAM [29] from English.

### 4.1 KHATT dataset

The KHATT dataset [48] was prepared by a research group at King Fahd University of Petroleum and Minerals (KFUPM). The KHATT dataset consists of 1000 handwritten forms written by 1000 distinct writers from different countries, and each writer contributed four paragraphs. The first and fourth paragraphs are fixed text, while the second and third paragraphs were selected uniquely from 46 information sources. In other words, the second and third paragraphs have different textual content for each writer, while the first and fourth contain the same text content for all writers. The designer of this dataset carefully selected the words and letters of the fixed paragraphs’ text so that they contain all possible forms of Arabic letters in both cursive and separated writing manners.

This paper kept the complete set of KHATT samples, and because the method is text-independent writer identification, one of the two fixed paragraphs is discarded to save the credibility of the results. Hence, paragraphs’ images were divided into two sets, the first containing one of the unique paragraphs and one of the fixed paragraphs, which are used in the training step, and the other containing one of the unique paragraphs is used for system testing.

### 4.2 IAM dataset

The IAM dataset [29] is an English dataset in which 657 writers wrote a different number of pages with different lengths from one writer to another. Some writers wrote full pages, while others wrote a few lines. This research adopts the IAM modifications done by [22, 49]. They chose two random handwritten pages for contributors who had more than two pages, and divided the page strictly in half for writers who had only one page. As a result of their preparation, the IAM dataset used in this experiment involves lowercase handwritten pages for 650 writers, with two samples for each writer. One is used to train the system, and the other is used for system testing.

## 5 Experimental results

The datasets used for conducting the experiments were introduced in the previous section. The identification rate achieved by this method depends on the values of two main groups of parameters for each of the proposed features: 1) Key-internal parameters and 2) Common parameters.

As an example of key-internal parameters, during the CPCA feature extraction, experiments show that the length of the line segment *Є* and the angular quantization *M* have direct impacts on the identification rate achieved by this feature. Furthermore, during the CON^3^ feature extraction, we found direct impacts for other parameters on the results achieved using this feature. These parameters are the angular quantization of both *θ*_*1*_ and *θ*_*2*_, length of line segment *Є*, distance quantization of the perpendicular distance and length quantization of the line connecting the endpoints of the curve fragment. Additionally, parameters *NP* and *GAP* have been found to have impacts on the identification rates of the two proposed features. All these parameters were tested and selected empirically for each feature on each dataset. Finally, some literature studies [6, 8–10, 50, 51] reported that the χ^2^, Euclidean and Manhattan metrics have achieved close results without providing evidence for the superiority of specific metrics. Therefore, this experiment uses the χ^2^ metric because it is widely used in many applications with the same background. The analytical results of the variations of the key-internal parameters are presented in section 5.1.

Regarding the common parameters, these parameters are common among all writer identification systems as this group of parameters are related to the task itself. This group of common parameters includes the size of the codebook, the amount of available text in the handwritten document and the number of scribes involved in the experiments. Section 5.2 presents the analytical results of the variations of common parameters.

In this experiment, multiclass SVM and nearest neighbor (NN) methods are used as classifiers. With nearest neighbor, the proposed method computes the distance between the feature vector of the query handwritten document and all the features vectors of the reference base and selects the writer of the handwritten document based on the minimum distance. The nearest neighbor is a simple classifier that is effective, does not need to be trained and does not need human intervention [52]. The proposed method implements the multiclass SVM using the one-against-all technique [53]. The SVM depends on a selected kernel function to perform complex data transformations, which in turn are used to maximize the separation boundaries between data points based on their labels or classes. A radial basis kernel function (RBF) is considered to train the SVM model, and the regularization parameter *C* is set to 1 for all classifiers.

### 5.1 Key-internal parameters analysis

This section presents the findings of a set of experiments to evaluate the impacts of the internal parameters of the proposed CPCA and CON^3^ features on the writer identification performance on both IAM and KHATT datasets. As stated in sections 3.1.1 and 3.1.2, the feature descriptors comprise some key parameters like contour fragment length *NP*, the distance between the starting points of each two successive contour fragments *GAP*, line fragments of length *Є*, the quantization of the contour points’ angles *N*_*a*_ and *M*. Additionally, the proposed CON^3^ feature comprises two additional parameters: (i) The perpendicular line distance quantization (*N*_*q*_) and (ii) The distance quantization of the line (*L*_*p*_) into *N*_*l*_.

**Fig 5:**
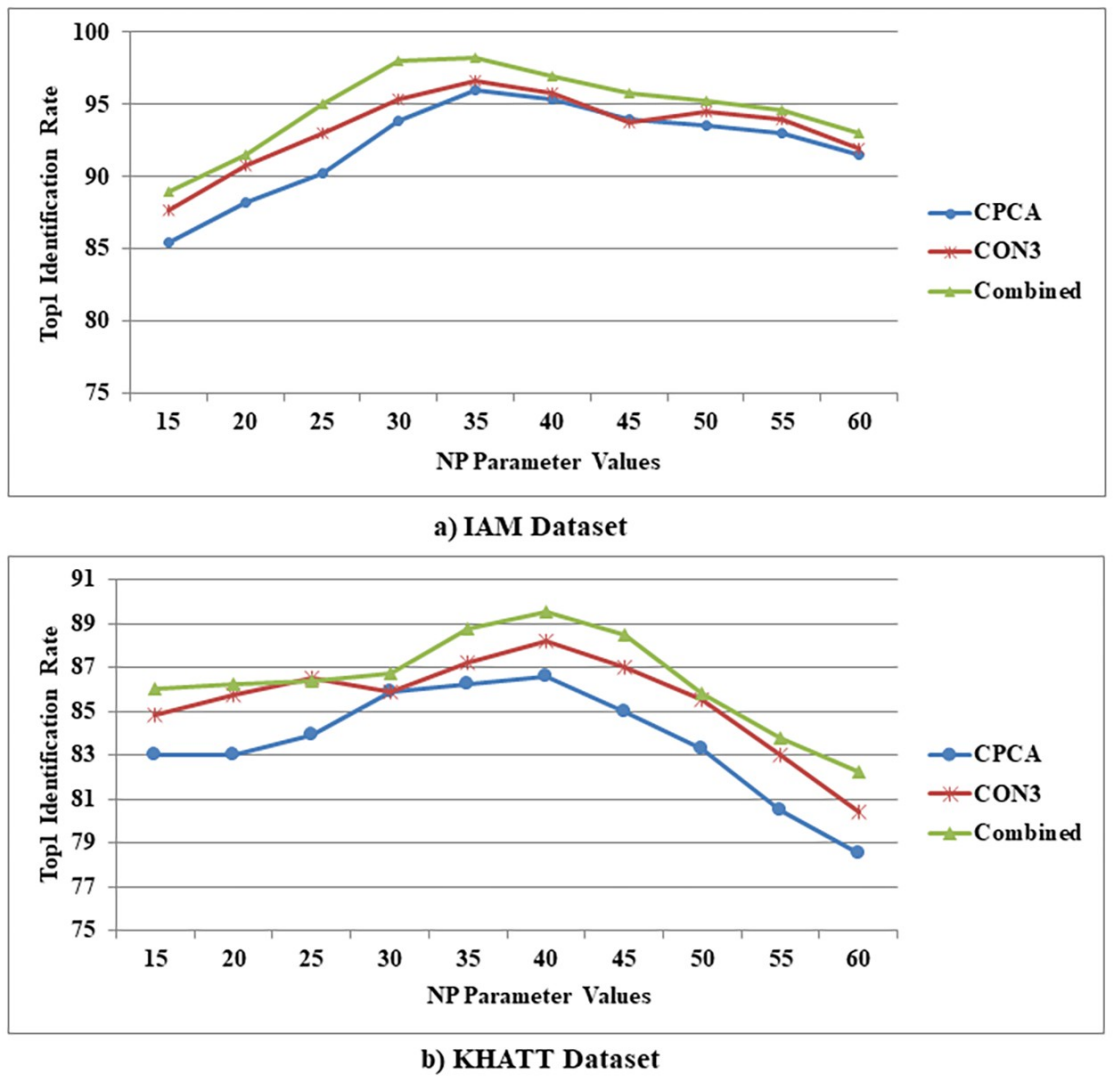
Writer identification with respect to contour fragment length on IAM and KHATT datasets.

**Fig 6:**
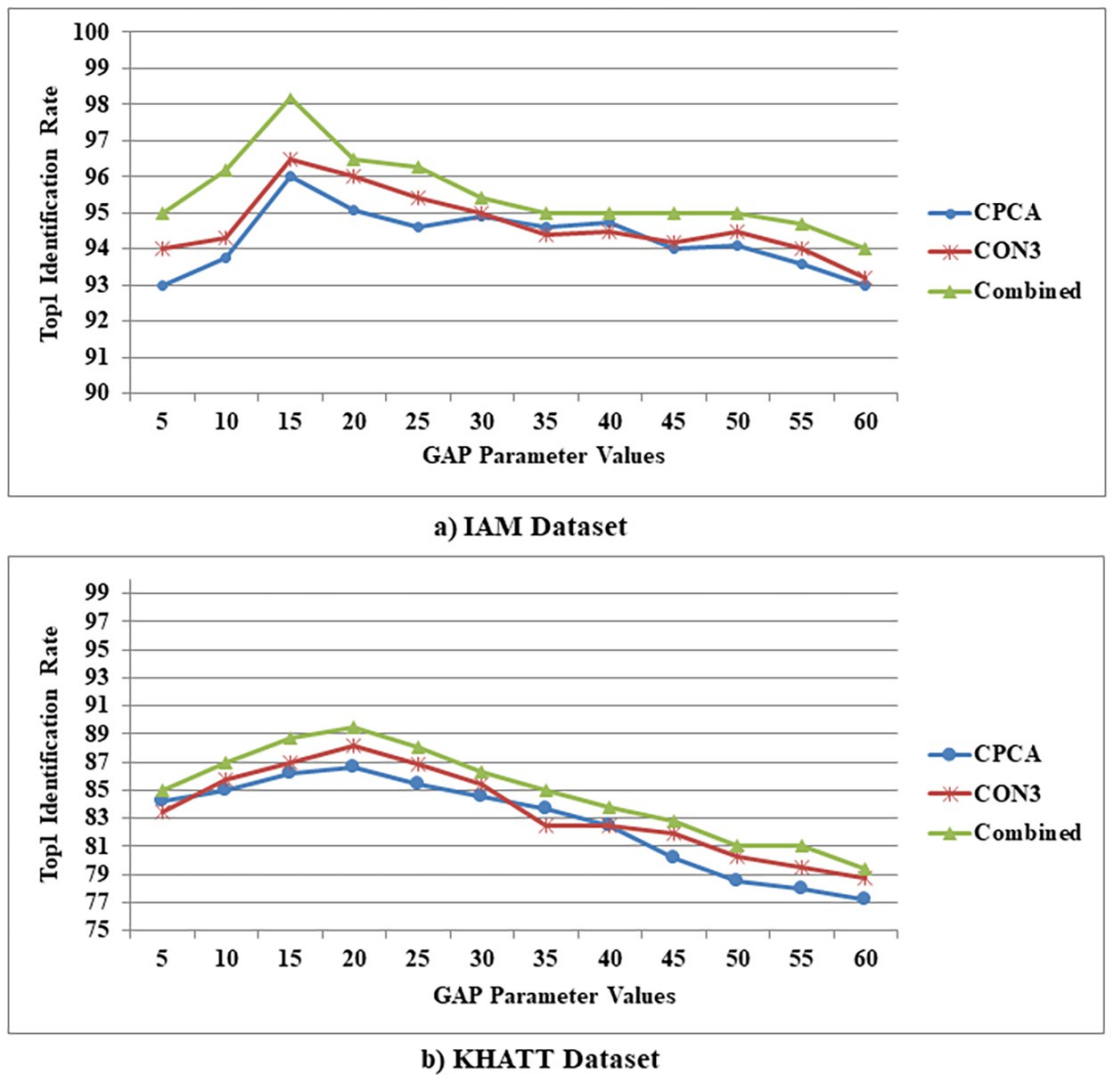
Writer identification with respect to GAP parameter on IAM and KHATT datasets.

**Fig 7:**
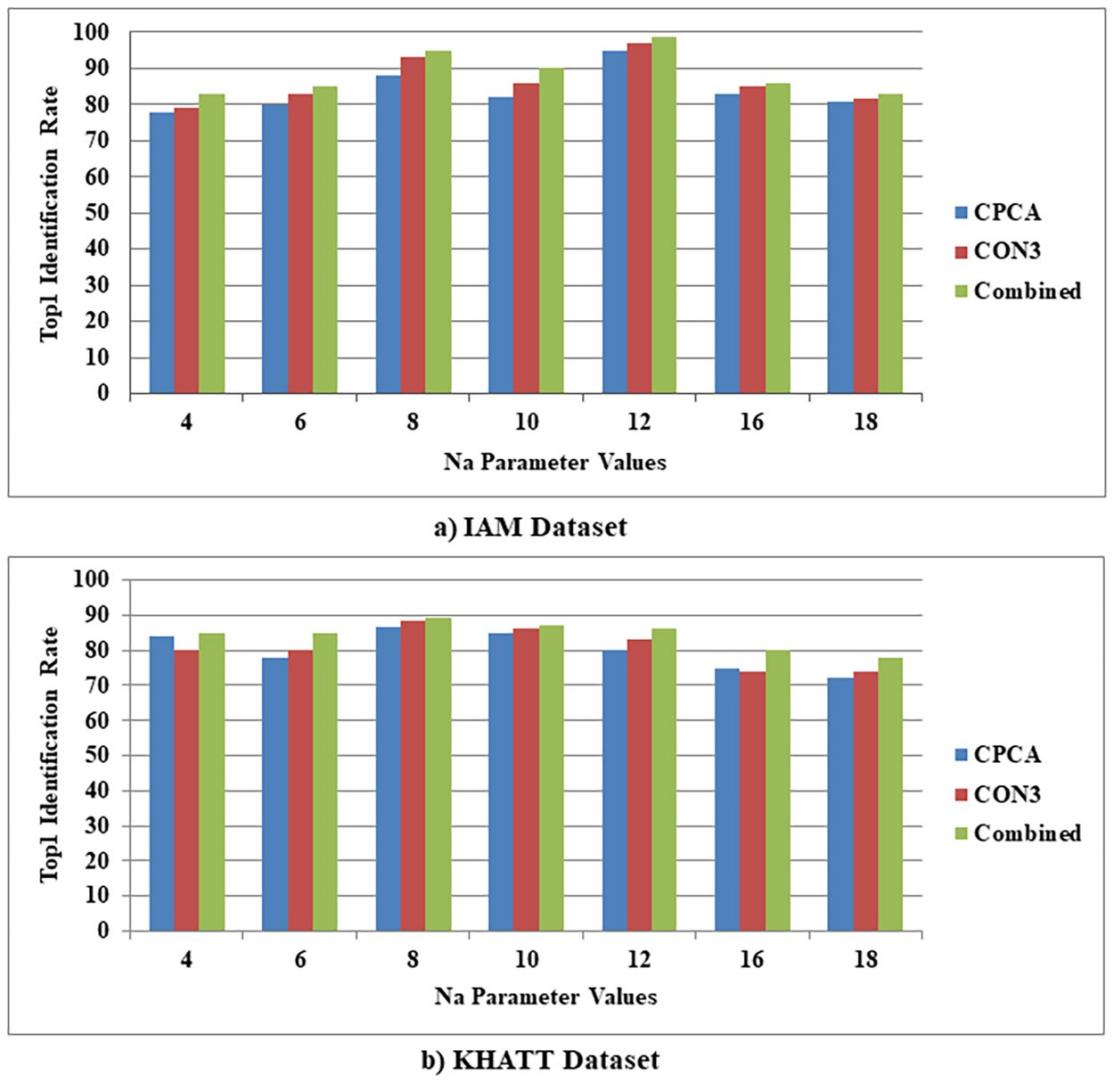
Writer identification with respect to angular quantization on IAM and KHATT datasets.

**Fig 8:**
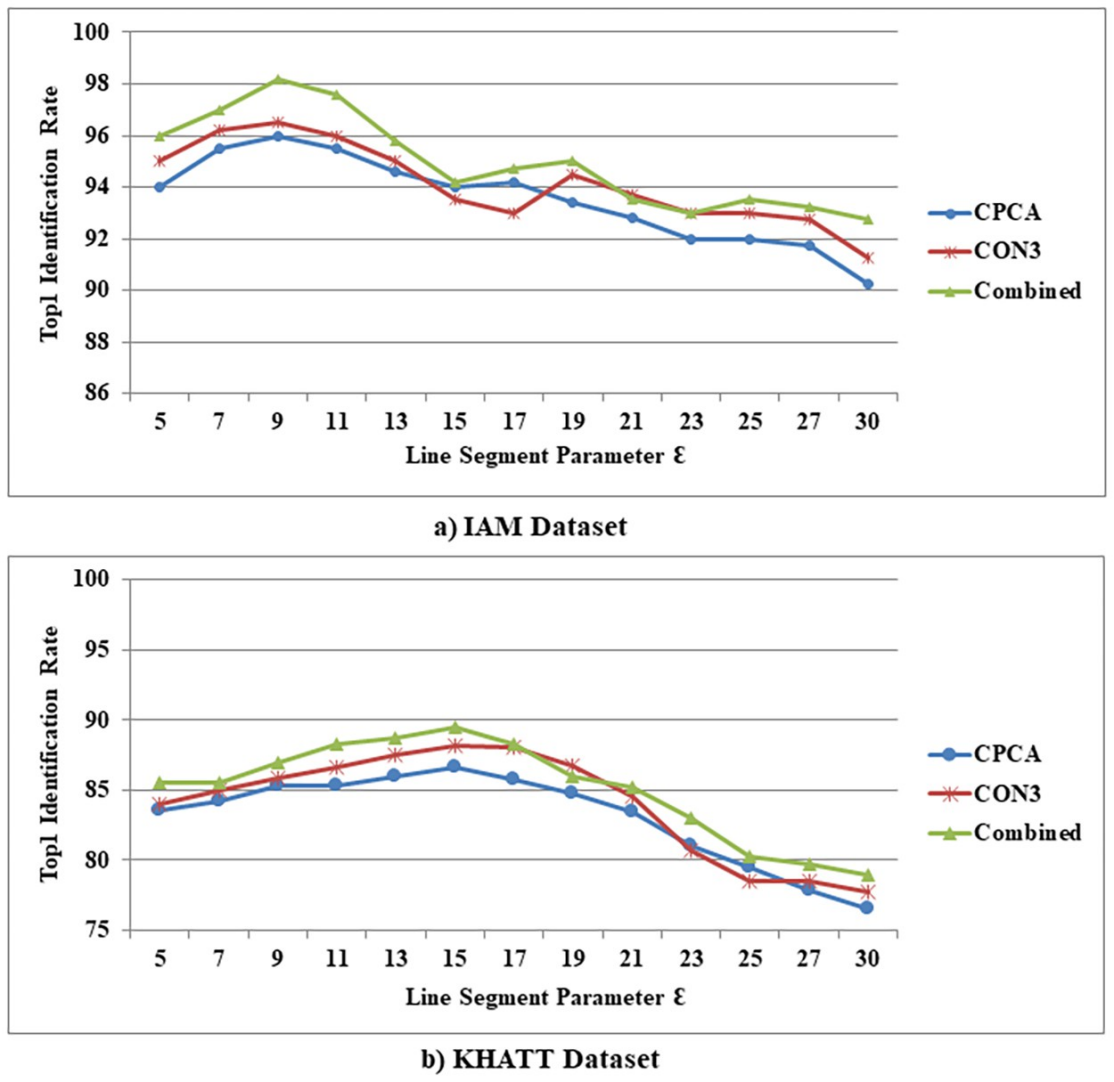
Writer identification with respect to line segment length on IAM and KHATT datasets.

The results reported in Tables 2–7 correspond to the optimal values of these parameters that are specified empirically for each dataset through a series of experiments. The parameters *NP = 35*, *GAP = 15*, *Na = 12*, *M = 8*, *Ɛ = 9*, *Nq = 10*, and *N*_*l*_ *= 12* of both CPCA and CON^3^ features are set to the optimal values to achieve the highest identification rate of 96% and 96.3% on IAM dataset using CPCA and CON^3^, respectively. Similarly, with KHATT dataset, the highest identification rate is achieved when the parameters of CPCA and CON^3^ features are set to the optimal values as follows: *NP = 40*, *GAP = 20*, *N*_*a*_ *= 8*, *M = 6*, *Ɛ = 15*, *Nq = 14*, and *N*_*l*_ *= 12* to achieve the highest identification rate of 86.6% and 88.2% using CPCA and CON^3^, respectively. From the definition of the proposed CPCA and CON^3^ features, there is a correlation between the *NP* and *GAP* parameters. Therefore, the identification rate is recorded for each pair values of (*NP*, *GAP*) as follows; for both IAM and KHATT datasets, the *GAP* parameter is set from 5 to the value of the parameter *NP* for each setting of the parameter *NP*. From Figs 5(a) and 6(a), it can be seen that the highest identification rate on IAM dataset is achieved with settings *NP = 35* and *GAP = 15* and this observation is consistent for CPCA, CON^3^ and their combination. On the other hand, Figs 5(b) and 6(b) show that the highest identification rate on KHATT dataset is achieved with parameter values set to *NP = 40* and *GAP = 20* for both CPCA, CON^3^ and their combination. By fixing the previous two parameters to their optimal values and continue with testing the influences of the other parameters on the overall system performance, we found significant impact of the angular quantization parameter *N*_*a*_ of the two proposed CPCA and CON^3^ features on both IAM and KHATT datasets. Fig 7 shows that the proposed method achieved the highest identification rate when the parameter value *N*_*a*_ is set to 12 and 8 on IAM and KHATT datasets. It is worthy here to mention that we fix the optimal settings of the parameters checked so far in order to check the remaining parameters in the successive experiments. As shown in Fig 8, the line segment parameter (*Ɛ*) values (*Ɛ = 9* and *Ɛ = 15*) prove to be the best setting on IAM and KHATT datasets. Additionally, the proposed CPCA and CON^3^ and their combination performed consistently across the two datasets.

**Fig 9:**
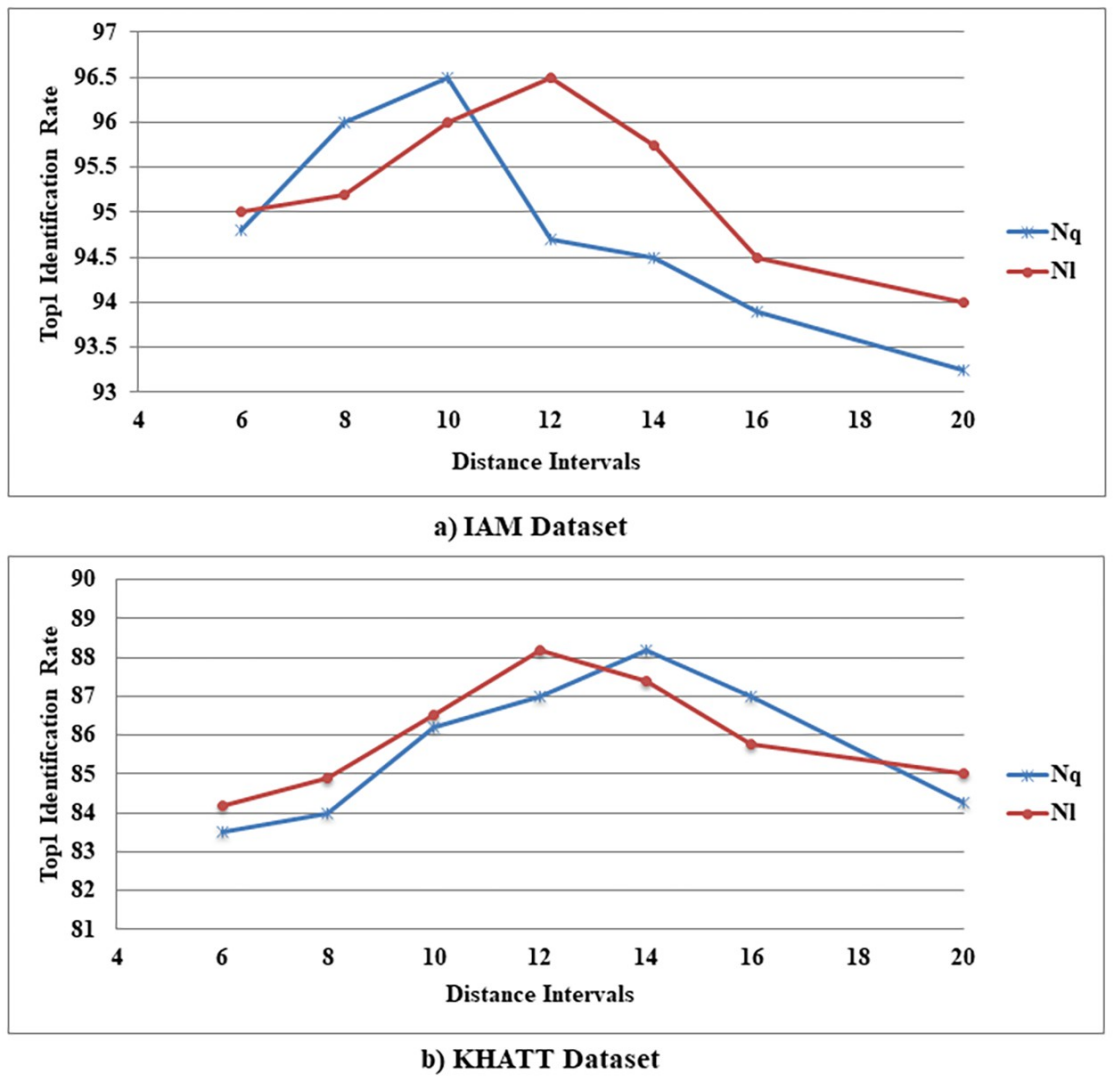
Writer identification with respect to distance quantization parameters N_q_ and N_l_ on IAM and KHATT datasets.

**Fig 10:**
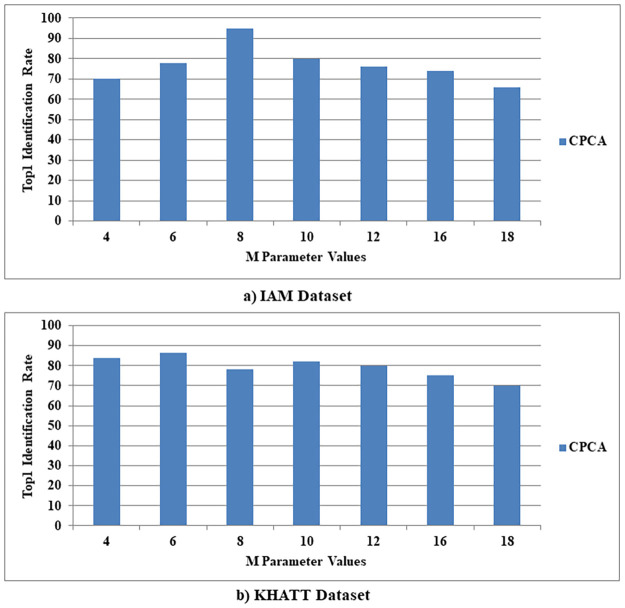
Writer identification with respect to Ø angle quantization on IAM and KHATT datasets.

As stated in section 3.1.2, the proposed CON^3^ feature is augmented by the distance quantization of the perpendicular line *P*_*d*_ and the line *L*_*p*_. The experiments involved the evaluation of possible impacts of these two parameters on the overall performance of the proposed system. Fig 9(a) shows that the system achieved the highest identification rate using CON^3^ feature when the parameters values *N*_*q*_ and *N*_*l*_ are set to 10 and 12, respectively on IAM dataset. Likewise, Fig 9(b) shows that the highest identification rate on KHATT dataset is achieved when the parameters values of *N*_*q*_ and *N*_*l*_ are set to 14 and 12, respectively. Additionally, section 3.1.1 detailed the definition of the CPCA feature. One of this feature attributes is the point’s curve angle Ø which is calculated for each point of the contour segment. The resultant angles are then quantized into angular quantization *M* which is evaluated to check its possible impact on the overall identification rate of the system. Fig 10 shows that the system achieved the highest identification rate using CPCA feature when the parameter *M* is set to 8 and 6 on IAM and KHATT datasets.

These internal-key parameters are critical. Their setting is user-specified and subject to the dataset and its configurations. As an example, we show in section 3.1.1 that the user can control the number of the extracted features using the *NP* and *GAP* parameters which we think is useful especially in case the available handwritten text is limited. However, such settings may lead to some deterioration of the overall system performance due to the variation of the number of extracted features.

### 5.2 Common parameters analysis

As we mentioned above, the common parameters are not related to a specific writer identification method, but they are related to all systems in the writer identification domain. The next sub-sections discuss the details of the possible impacts of these parameters on the overall performance of the proposed writer identification system.

#### 5.2.1 Sensitivity to the amount of available text

This method conducted a series of runs to evaluate the possible impacts of the amount of available text in a handwritten document for both training and testing sets. The available text varies from one writer’s document to another writer’s document. Hence, the experiments were performed for only a subset of writers, namely, 100 writers who had at least 45 and 30 connected components in the training and testing sets, respectively. The system uses the nearest neighbor classifier, and the codebook size is set to 100 during this series of runs.

The intention here is to evaluate the possible impacts of the amount of available text on the overall system performance. Therefore, the system starts with 45 connected components for the training and 30 connected components for the testing. The number of connected components is then increased gradually for both training and testing. It is worth mentioning that we fixed the number of connected components instead of fixing the number of words, as different words can lead to different numbers of connected components. Table 1 presents the results of this experiment on both the IAM and KHATT datasets using the proposed CPCA and CON^3^ features, and their combination. As expected, the more available text, the better the identification rate is. A higher identification rate was observed in the case where 90 and 60 connected components were considered in training and testing, respectively. As presented in Table 1, the proposed method achieved good results even with a small size of training and testing text, especially on the IAM dataset. However, one can easily note that there is a relatively large gap in the scored results between the case of considering a small amount of text and a large amount of text within the KHATT dataset. This observation is natural and confirms the correctness of the literature observation about the complexity of the Arabic language.

**Table 1:** The impacts of the available handwritten text.

Dataset Name	Feature Name	Conn comps of known writer document / Conn comps of query document		
		45/30	60/40	90/60
**IAM**	CPCA	93%	95%	98%
	CON^3^	94%	96%	99%
	Combined	94%	98%	100%
**KHATT**	CPCA	72%	83%	90%
	CON^3^	78%	87%	93%
	Combined	79%	87%	95%

#### 5.2.2 Sensitivity to the number of writers

**Fig 11:**
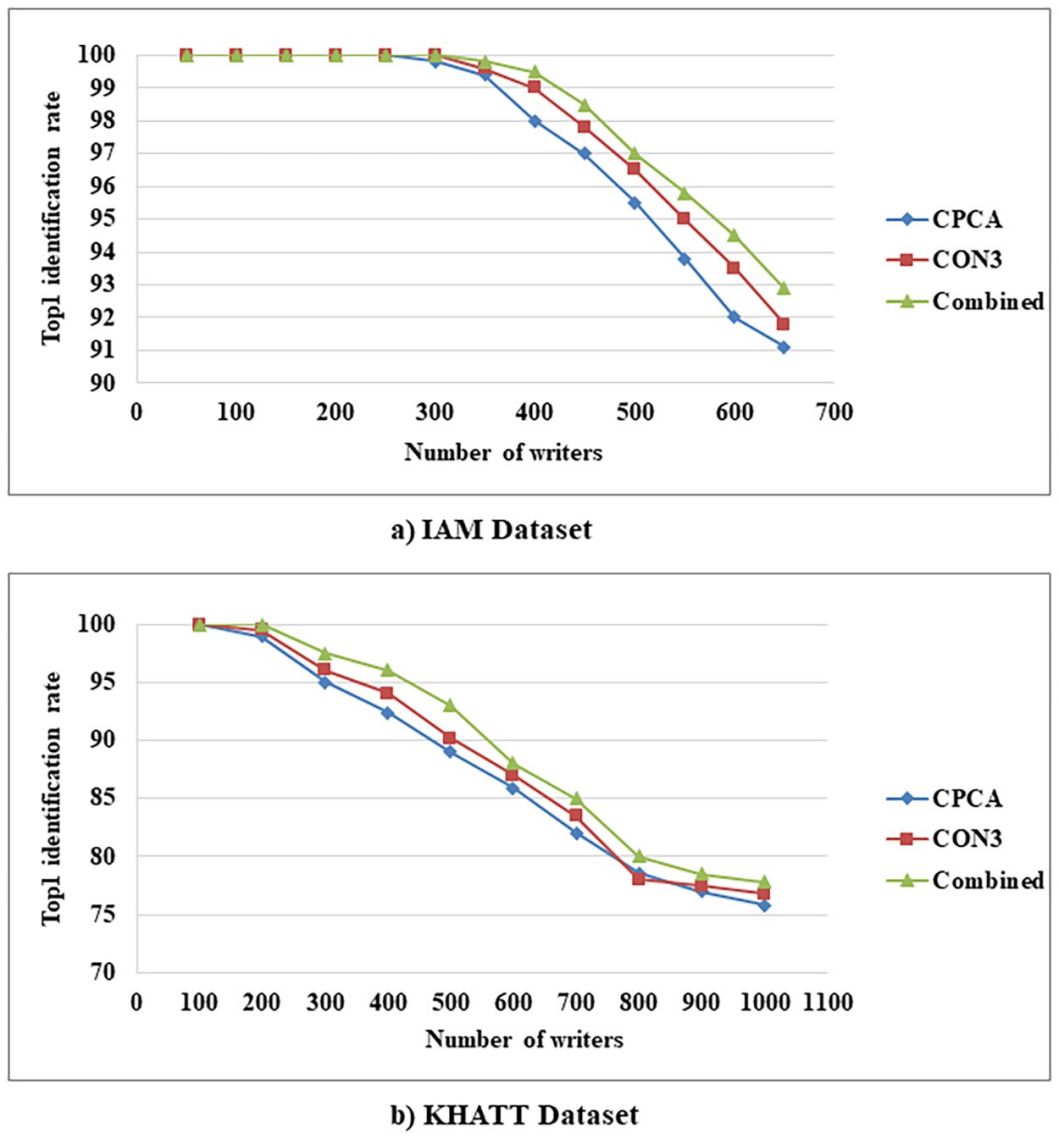
Writer identification with respect to the number of writes using NN classifier on IAM and KHATT datasets.

**Fig 12:**
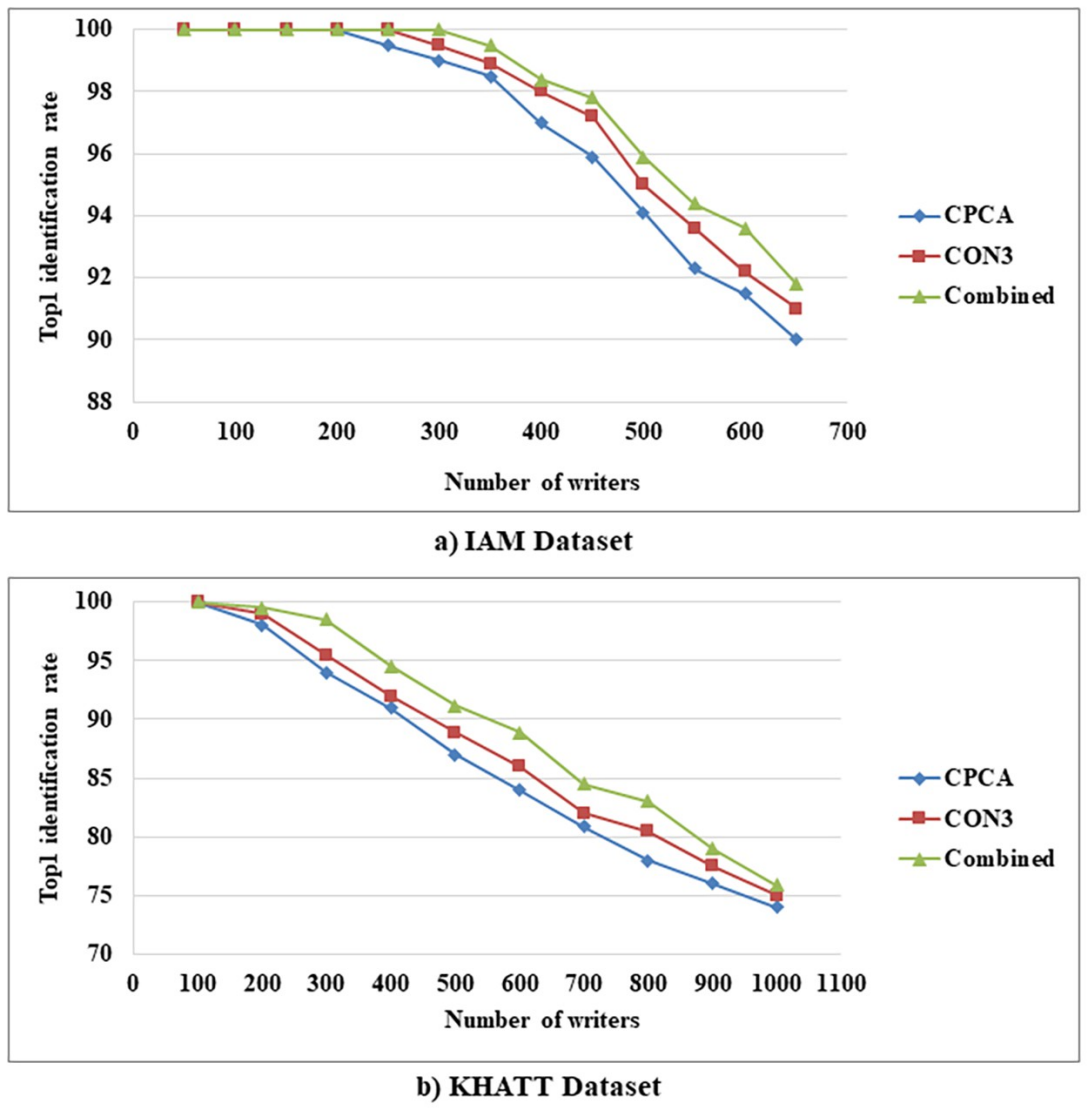
Writer identification with respect to the number of writes using SVM classifier on IAM and KHATT datasets.

Regarding the parameter of the number of writers, which reflects the stability of the system, this method conducted a series of runs to evaluate the impact of this parameter on the overall system identification rate. The experiment is done by changing the number of writers in each run. The system starts by considering 50 writers from the IAM dataset and then increases the number of writers by 50 writers in successive runs until the total number of writers in the database is reached. The same strategy is done for the KHATT dataset but considering 100 writers in the first run and an increment of 100 writers in the successive runs. The codebook size is set to 200 during this experiment, and the resulting identification rates on the IAM and KHATT datasets using both classifiers are shown in Figs 11 and 12. With the IAM dataset, slight degradation is observed in the scored identification rate, which is a natural behavior for writer identification systems, as reported in [11]. However, the proposed method achieved stable performance when considering the small differences between the identification rate on a small set of writers and the whole set of IAM writers.

The identification rate starts at 100% for the case of 50 scribes and deteriorates slightly as the scribes increase to 92.9% for the case with the complete set of IAM writers using the combination of CPCA and CON^3^ features. The situation is different for the KHATT dataset, it can be seen from Fig 11(b) that the performance deterioration is relatively large, starting at 100% for the case of 100 writers and deteriorating to 77.8% for the case with the complete set of KHATT writers using the combination of CPCA and CON^3^ features. This observation is very natural, as it confirms the literature belief [11] that the Arabic language is more complex than the English language. Additionally, this observation confirms the fact that the higher number of writers and the higher content variety lead to more complexity and less system performance. It can be observed that the system did not achieve the best identification rate during this experiment. This is because we did not apply the optimal values for some parameters, such as codebook size. These parameters need to be varied each time the dataset or the number of writers of the dataset is changed.

### 5.3 Sensitivity to the codebook size

The codebook size has been proven to have a large impact on both the identification rate and the efficiency of writer identification systems [13]. According to this literature observation, the proposed method conducted a series of runs to evaluate the impact of the codebook size on the overall performance of the system.

**Fig 13:**
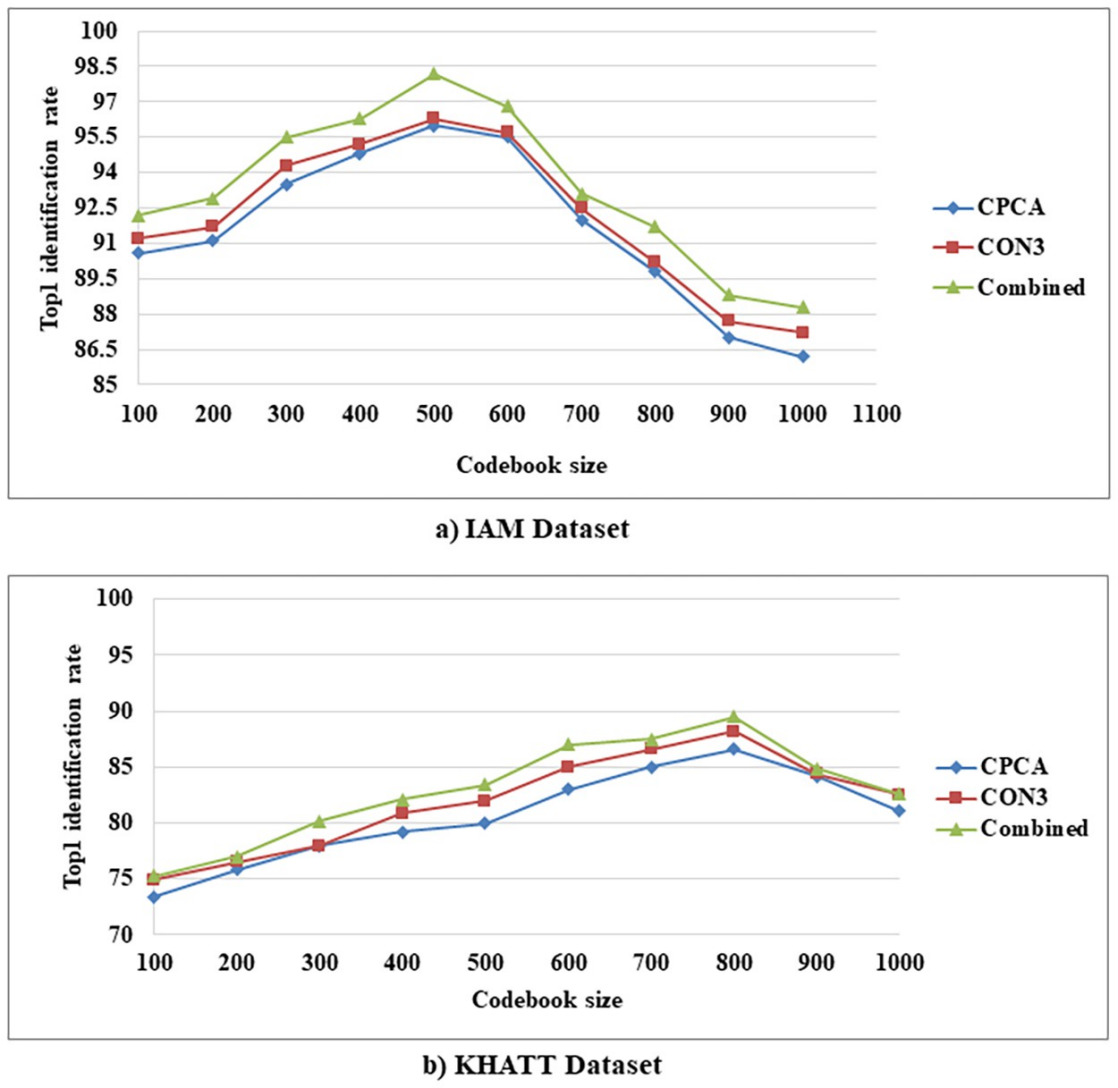
Writer identification with respect to the codebook size using NN classifier on IAM and KHATT datasets.

**Fig 14:**
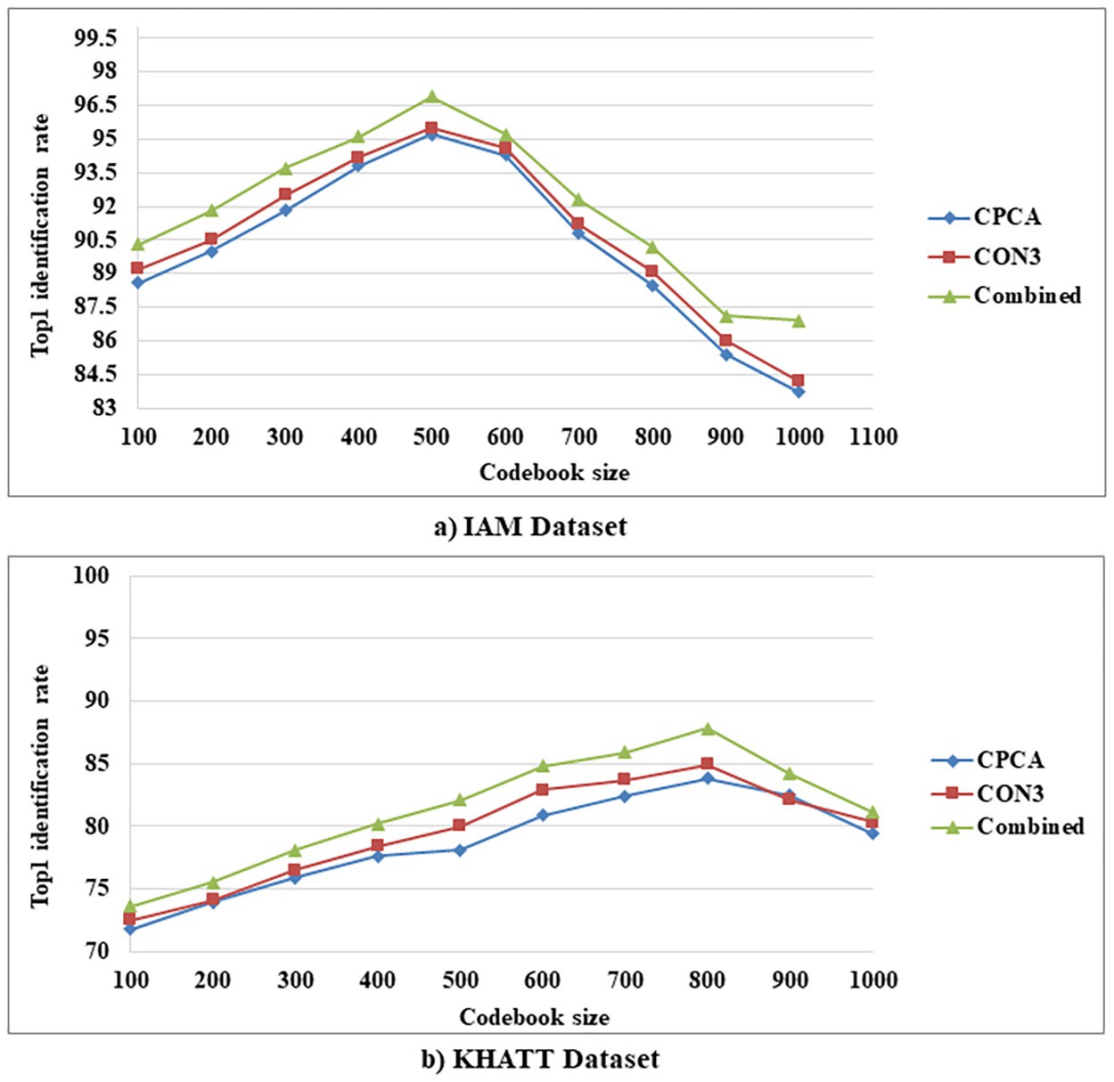
Writer identification with respect to the codebook size using SVM classifier On IAM and KHATT datasets.

The system starts with 100 codewords, and then the number of codewords is increased by 100 in each successive run. The codebook size incrementing is stopped when the system reaches 1000 codewords. Tables 2–7 present the identification rate of the proposed method on both IAM and KHATT datasets using both nearest neighbor and SVM classifiers. It is clear that the codebook size parameter has a large impact on the scored identification rate, and the system achieved the highest rate when the codebook size was set to 500 and 800 on the IAM and KHATT datasets, respectively. An interesting observation can be seen from Tables 2–7. The nearest neighbor classifier achieved better performance than the support vector machine in all cases on both the IAM and KHATT datasets, and this observation confirms the superiority of the nearest neighbor classifier over other classifiers, which was reported by [52]. Additionally, the CPCA and CON^3^ features, and their combination achieved the highest identification rate with the same codebook size on the same dataset, and this observation is consistent on the IAM and KHATT datasets using both nearest neighbor and support vector machine classifiers, as depicted in Figs 13 and 14.

**Table 2:** CPCA results on IAM dataset.

Codebook size	Classifier	Top1%	Top5%	Top7%	Top10%
100	Nearest-Neighbor	90.6	92	93.5	95.5
	SVM	88.6	90.3	90.3	91.1
200	Nearest-Neighbor	91.1	92.8	92.8	96.2
	SVM	90	91.1	91.4	91.5
300	Nearest-Neighbor	93.5	94.2	94.9	97.8
	SVM	91.8	92.2	92.2	92.5
400	Nearest-Neighbor	94.8	94.9	95.5	96.3
	SVM	93.8	94.8	94.8	95.1
**500**	Nearest-Neighbor	**96**	**97.5**	**98.2**	**98.9**
	SVM	**95.2**	**95.8**	**95.8**	**96.2**
600	Nearest-Neighbor	95.5	96.9	96.9	97.1
	SVM	94.3	94.5	94.5	94.5
700	Nearest-Neighbor	92	93.5	94	94.9
	SVM	90.8	91.2	91.4	91.8
800	Nearest-Neighbor	89.7	89.7	91.1	92
	SVM	88.5	88.8	89.1	90
900	Nearest-Neighbor	86.9	88	88.6	89.7
	SVM	85.4	86.9	87.2	88.8
1000	Nearest-Neighbor	86.2	88	88.9	90.6
	SVM	83.7	84.3	85.7	85.8

**Table 3:** CON^3^ results on IAM dataset.

Codebook size	Classifier	Top1%	Top5%	Top7%	Top10%
100	Nearest-Neighbor	91.2	93.1	93.3	96.3
	SVM	89.2	91.4	91.4	91.1
200	Nearest-Neighbor	91.7	93.2	93.7	93.7
	SVM	91	91.1	91.7	92.3
300	Nearest-Neighbor	94.3	95.1	95.4	95.7
	SVM	92.5	93.4	93.5	93.8
400	Nearest-Neighbor	95.2	96.1	96.3	96.3
	SVM	94.2	95.3	95.5	95.9
**500**	Nearest-Neighbor	**96.3**	**97.9**	**98.6**	**99.1**
	SVM	**95.5**	**96**	**96.2**	**96.4**
600	Nearest-Neighbor	95.7	96.2	96.2	96.2
	SVM	94.6	94.8	94.8	95.2
700	Nearest-Neighbor	92.5	92.9	93.4	93.7
	SVM	91.2	91.7	91.8	91.8
800	Nearest-Neighbor	90.2	90.6	90.8	91.1
	SVM	89.1	89.2	89.5	89.7
900	Nearest-Neighbor	87.7	88.6	88.6	88.8
	SVM	86	86.7	86.8	87.1
1000	Nearest-Neighbor	87.2	87.7	88	88.5
	SVM	84.2	84.6	84.8	85.4

**Table 4:** COMBINED FEATURES results on IAM dataset.

Codebook size	Classifier	Top1%	Top5%	Top7%	Top10%
100	Nearest-Neighbor	92.2	93.5	94.3	94.6
	SVM	90.3	92.3	92.6	92.8
200	Nearest-Neighbor	92.9	93.5	93.8	94.3
	SVM	91.8	92.9	93.2	93.5
300	Nearest-Neighbor	95.5	96.3	96.8	96.8
	SVM	93.7	94.5	94.8	95.1
400	Nearest-Neighbor	96.3	96.9	97.2	97.4
	SVM	95.1	96	96.3	96.6
**500**	Nearest-Neighbor	**98.2**	**98.6**	**98.8**	**98.9**
	SVM	**96.9**	**97.5**	**97.8**	**97.8**
600	Nearest-Neighbor	96.8	97.4	97.7	98
	SVM	95.2	95.7	96	96.3
700	Nearest-Neighbor	93.1	93.4	93.4	93.7
	SVM	92.3	93.1	93.2	93.4
800	Nearest-Neighbor	91.7	92.2	92.5	92.5
	SVM	90.2	90.8	91.2	91.7
900	Nearest-Neighbor	88.8	89.4	90	90.3
	SVM	87.1	88.2	88.8	88.8
1000	Nearest-Neighbor	88.3	88.8	89.2	89.7
	SVM	86.9	87.5	88.2	88.9

**Table 5:** CPCA results on KHATT dataset.

Codebook size	Classifier	Top1%	Top5%	Top7%	Top10%
100	Nearest-Neighbor	73.4	74.2	75	76.3
	SVM	71.8	72.7	73.1	74.3
200	Nearest-Neighbor	75.8	76.4	78	79.1
	SVM	73.9	75.1	75.3	76.2
300	Nearest-Neighbor	78.1	78.5	78.5	79.7
	SVM	75.9	77	77.5	78.1
400	Nearest-Neighbor	79.2	80.1	81	82.7
	SVM	77.6	78.9	79.2	79.9
500	Nearest-Neighbor	80	81.4	81.9	83.1
	SVM	78.1	79	79.1	80.3
600	Nearest-Neighbor	83	84.2	85.3	85.9
	SVM	80.9	82.5	83.6	84.1
700	Nearest-Neighbor	85.2	85.9	86	86.8
	SVM	82.4	83.6	84.1	84.6
**800**	Nearest-Neighbor	**86.6**	**87.5**	**89.1**	**92.2**
	SVM	**83.8**	**84.2**	**85.1**	**86.9**
900	Nearest-Neighbor	84.2	84.7	85.9	88.5
	SVM	82.5	83	83.3	83.8
1000	Nearest-Neighbor	81.1	83.5	84.8	86.7
	SVM	79.4	81.3	82.2	82.9

**Table 6:** CON^3^ results on KHATT dataset.

Codebook size	Classifier	Top1%	Top5%	Top7%	Top10%
100	Nearest-Neighbor	74.9	75.8	76.9	77.4
	SVM	72.5	73.9	74.3	75.7
200	Nearest-Neighbor	76.5	77.1	78	79.1
	SVM	75	75.4	75.9	76.8
300	Nearest-Neighbor	78	78.8	78.8	79.7
	SVM	76.5	77.3	78.2	78.9
400	Nearest-Neighbor	80.9	81.5	83	83.7
	SVM	78.4	79.3	79.7	81
500	Nearest-Neighbor	82	82.4	82.9	84.1
	SVM	80	80.6	81.2	81.8
600	Nearest-Neighbor	85	86.2	87.1	87.9
	SVM	82.9	84	84.3	85.4
700	Nearest-Neighbor	86.6	87.1	88	88.8
	SVM	83.7	84.9	85.1	86.3
**800**	Nearest-Neighbor	**88.2**	**89.3**	**89.9**	**92.2**
	SVM	**84.9**	**86.2**	**86.8**	**88.1**
900	Nearest-Neighbor	84.4	84.4	85.2	87.9
	SVM	82.1	83.3	83.3	84.1
1000	Nearest-Neighbor	82.5	84	84.8	85.3
	SVM	80.3	81	81.9	83.2

**Table 7:** COMBINED FEATURES results on KHATT dataset.

Codebook size	Classifier	Top1%	Top5%	Top7%	Top10%
100	Nearest-Neighbor	75.3	76	76.9	76.9
	SVM	73.6	75.1	76.7	76.7
200	Nearest-Neighbor	77.8	77.9	78	79.4
	SVM	75.5	76.8	77.2	77.9
300	Nearest-Neighbor	80.2	80.8	80.8	81.7
	SVM	78.1	79.3	79.8	80.1
400	Nearest-Neighbor	82.1	82.5	83.9	84.2
	SVM	80.2	81.1	81.7	82.4
500	Nearest-Neighbor	83.4	83.8	84	84.8
	SVM	82.1	83.3	83.6	83.9
600	Nearest-Neighbor	87	87.8	87.9	88.5
	SVM	84.8	85.7	86.2	87.1
700	Nearest-Neighbor	87.4	87.4	87.4	88.8
	SVM	85.9	86.8	86.9	87.4
**800**	Nearest-Neighbor	**89.5**	**90**	**91.6**	**92.9**
	SVM	**87.8**	**88.6**	**89.2**	**90.7**
900	Nearest-Neighbor	84.9	85.4	85.8	87.1
	SVM	84.2	84.8	85.1	86.2
1000	Nearest-Neighbor	82.6	83.5	84.3	85.7
	SVM	81.1	82.3	82.9	84.8

### 5.4 Writer identification

Writer identification is the task of recognizing the writer of a query handwritten document by retrieving a candidate list of documents that are similar to the query document. This candidate list is ordered in increasing order based on the dissimilarity value to the query document. The hit list can be considered as part of the candidate list. This experiment adopted typical sizes of the hit list, which are 1, 5, 7, and 10. Accordingly, top1, top5, top7 and top10 represent the overall identification rate of each feature on the considered datasets. Top1 means that the proposed method counts correctly if and only if the document at the top of the hit list is written by the same writer of the query document. Similarly, for top5, top7, and top10, the method counts correctly if one of the top five, top seven, or top ten documents is written by the same writer of the query document. The scored writer identification rates using the nearest neighbor and support vector machine classifiers are presented in Tables 2–7. As discussed previously, the identification rate is expressed by the top1, top5, top7, and top10 measures. It can be noted that the identification rate is consistent for the CPCA and CON^3^ features, and their combination on the two datasets. The combination of CPCA and CON^3^ features achieved the best identification rate, reading 98.2% and 89.5% on the IAM and KHATT datasets, respectively. However, Tables 2, 3, 5 and 6 show that the system achieved good identification rates using the CPCA and CON^3^ features on the IAM and KHATT datasets.

This paper conducted a comparative study with dominant methods that have been proposed in the literature. However, we emphasize that it is quite difficult to compare specific work to other works for two main reasons. First, some works utilize datasets that are not publicly accessible. Second, even if the dataset is public, researchers often utilize it with different configurations. To ensure the fairness of the performance comparison of our system with the state-of-the-art systems on the writer identification task, we only considered those studies which evaluate their methods on the whole set of scribes of both IAM and KHATT datasets. It is worthy here to mention that we adopted the same configurations of IAM dataset used in [6]. Regarding the KHATT dataset, we excluded one of the two fixed paragraphs of each form as discussed in section 4.1 and the dataset is divided into 70% and 30% subsets for training and testing, respectively.

The top1 identification rate achieved by our system along with the current state-of-the-art systems on IAM and KHATT datasets is shown in Tables 8 and 9, respectively. Comparing the writer identification rates scored on the IAM dataset, the proposed method in this paper achieved 98.2%, which is the highest identification rate among the state-of-the-art methods including deep learning-based ones. The next top methods are the systems presented in [54, 55], both with top1 identification rates of 97.8%. Among the deep learning-based methods, the method proposed by [56] achieved the highest identification rate of 97.5% followed by the method proposed by [57] which scored an identification rate of 97.27%. From Table 8, one can easily observe that the handcrafted features-based systems surpassed those systems developed using deep learning techniques. This observation supports the conclusions of [34–36] and the 2016 International Conference on Pattern Recognition (ICPR2016) and the 2017 International Conference on Document Analysis and Recognition (ICDAR2017) competitions winners.

**Table 8:** Comparison to the state-of-the-art writer identification methods for IAM dataset.

Dataset	Features Type	# Writers	Methods	Identification Rate (Top1%)
**IAM dataset [29]**	Structural-based	650	Khalifa et al. [13]	92
	Textural-based	657	Hannad et al. [11]	89.54
	Structural-based	650	Bulacu and Schomaker [16]	89
	Grapheme-based	650	Siddiqi and Vincent [41]	91
	Textural-based	657	Lai et al. [62]	95.15
	Textural-based	650	Khan et al. [55]	97.8
	Grapheme-based	650	He and Schomaker [6]	89.9
	Structural-based	650	Ghiasi and Safabakhsh [17]	93.7
	Textural-based	657	Chahi et al. [30]	96.8
	Textural-based	657	Chahi et al. [61]	94.06
	Structural-based	657	Kumar and Sharma [54]	97.8
	Textural-based	657	Hadjadji and Chibani [51]	94.5
	Textural-based	657	Singh et. al. [59]	97.62
	Auto-learned	657	He and Schomaker [63]	96.6
	Auto-learned	657	He and Schomaker [64]	96.3
	Auto-learned	657	He and Schomaker [65]	69.5
	Auto-learned	650	Nguyen et. al. [66]	90.12
	Auto-learned	657	Javidi and Jampour [56]	97.5
	Auto-learned	657	Kumar and Sundaram [67]	93.55
	Auto-learned	657	Kumar and Sharma [57]	97.27
	Structural-based	650	**Proposed Method**	**98.2**

**Table 9:** Comparison to the state-of-the-art writer identification methods for KHATT dataset.

Dataset	Features Type	# Writers	Methods	Identification Rate (Top1%)
**KHATT dataset [40]**	Structural-based	1000	Djeddi et al. [26]	84.10
	Grapheme-based	1000	Bennour et. al. [33]	62.81
	Textural-based	1000	Christlein et. al. [14]	97.2
	Textural-based	1000	Singh et. al [59]	95.60
	Structural-based	1000	Ahmed et al. [68]	62.93
	Textural-based	1000	Asi et al. [69]	85.5
	Textural-based	1000	Abbas et al. [31]	77.1
	Textural-based	1000	Hannad et al. [70]	85.4
	Auto-learned	1000	Chammas et al. [71]	86.1
	Auto-learned	1000	**Christlein and Maier [58]**	**99.6**
	Auto-learned	828	Khosroshahi et al. [60]	99.74
	Structural-based	1000	Proposed Method	89.5

Few studies have been carried out on the KHATT dataset with comparison to those studies that have been performed on the IAM dataset. Table 9 summarizes state-of-the-art methods that considered the whole KHATT dataset, and the method proposed by [58] achieved the highest top1 identification rate reading 99.6% followed by another method [14] proposed by the same researchers (score of 97.2%). The next top method is the system presented in [59], with top1 identification rates of 95.6%. Our proposed method scored the 4^th^ position in the ranking of the state-of-the-art methods summarized in Table 9 if we excluded [60] as this study considered only 828 scribes out of 1000 scribes who contributed to the KHATT dataset. Interestingly, the three state-of-the-art methods that outperformed our proposed method seem not to consider a common practice in independent-text writer identification. That is, they did not indicate removing of fixed paragraphs in the KHATT dataset. In contrary, our proposed method belongs to the text-independent group, which necessitates the dataset configuration to exclude the repeated text. For this reason, we exclude one of the two fixed text paragraphs as mentioned in section 4.1. All methods in [14, 58, 59] which outperformed our proposed system on KHATT dataset do not refer to which group (text-dependent or text-independent) their systems belong, however, their dataset setup does not exclude the repeated text (fixed text paragraphs) which in turn leads to higher identification rate as reported by [30]. Furthermore, this approach-wise difference leads to the usage of different handwritten samples per writer which does not guarantee fair performance comparison [61]. Finally, we think that this drop might be because of the shortcoming that originated from the fragmentation step of the proposed method itself. This shortcoming causes some loss of information from the handwritten connected component contour. As mentioned in subsections 3.1.1 and 3.1.2, the proposed method segments the contour into segments of fixed length specified by the NP parameter starting by the most left point on the contour. This process continues in clockwise direction until the last point of the contour is reached. The method discards the last segment if its length is less than the NP parameter. This strategy causes loss of information from the connected component contour which may affect the ability of the system to accurately identify the query writers.

### 5.5 Misclassification errors analysis

**Fig 15:**
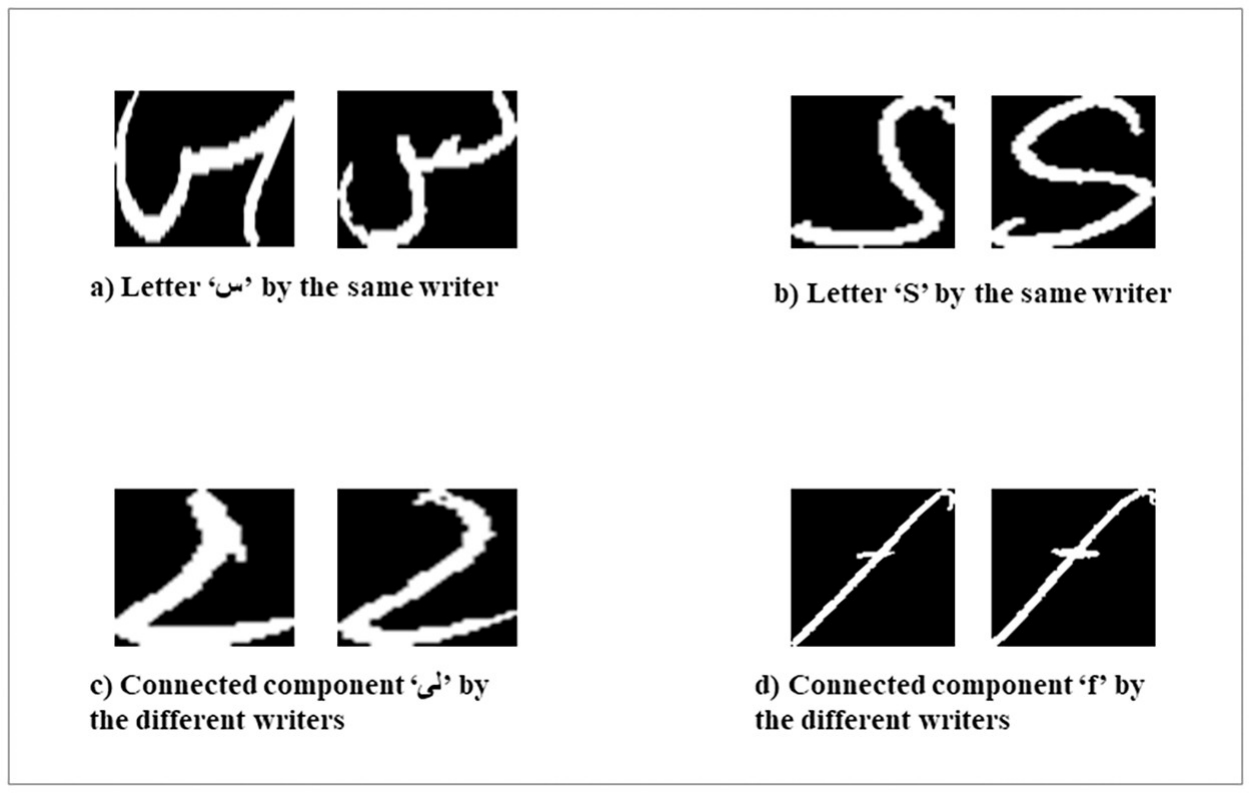
Proposed system misclassification errors.

The proposed method is subjected to some misclassification errors which are mainly due to two reasons: 1) the cases in which the same writer draws the same connected component /basic shapes in different ways. For example, Arabic language is one of the languages that have different writing styles in which the writer does not follow a specific style during his draft production. This leads to produce different shapes for the same connected component/basic shape which eventually affects the ability of the system to accurately classify the query writers. Examples of this case are shown in Fig 15. Fig 15(a) shows that the letter SIN ‘س’ is drawn differently by the writer from KHATT dataset and Fig 15(b) shows that the letter ‘S’ is drawn differently by the same writer from IAM datasets. 2) some connected components /basic shapes are drawn similarly by different writers which is one of the cases that the proposed method may misclassify the query writers. Fig 15(c) shows that the connected component ‘لى’ is drawn similarly by writer #575 and writer #137 from KHATT dataset and Fig 15(d) shows that the letter ‘F’ is drafted similarly by writer #2 and writer #21 from IAM dataset”.

### 5.6 Computation time analysis

The proposed writer identification approach has been implemented on AURORA R7 computer with an eighth-generation processor core I7, 3.2 GHz, and 16 GB RAM running the Windows 10 operating system. The system is developed using MATLAB R2015a^®^ platform.

A series of experiments have been done to empirically evaluate all parameters that we believe have possible impacts on the system performance and hence reporting the results obtained in this section. These extensive experiments impose the need for long execution time (days) due to two main reasons: (i) Considering the complete set of scribes of large scale datasets; (ii) the diversity of the experiments.

**Fig 16:**
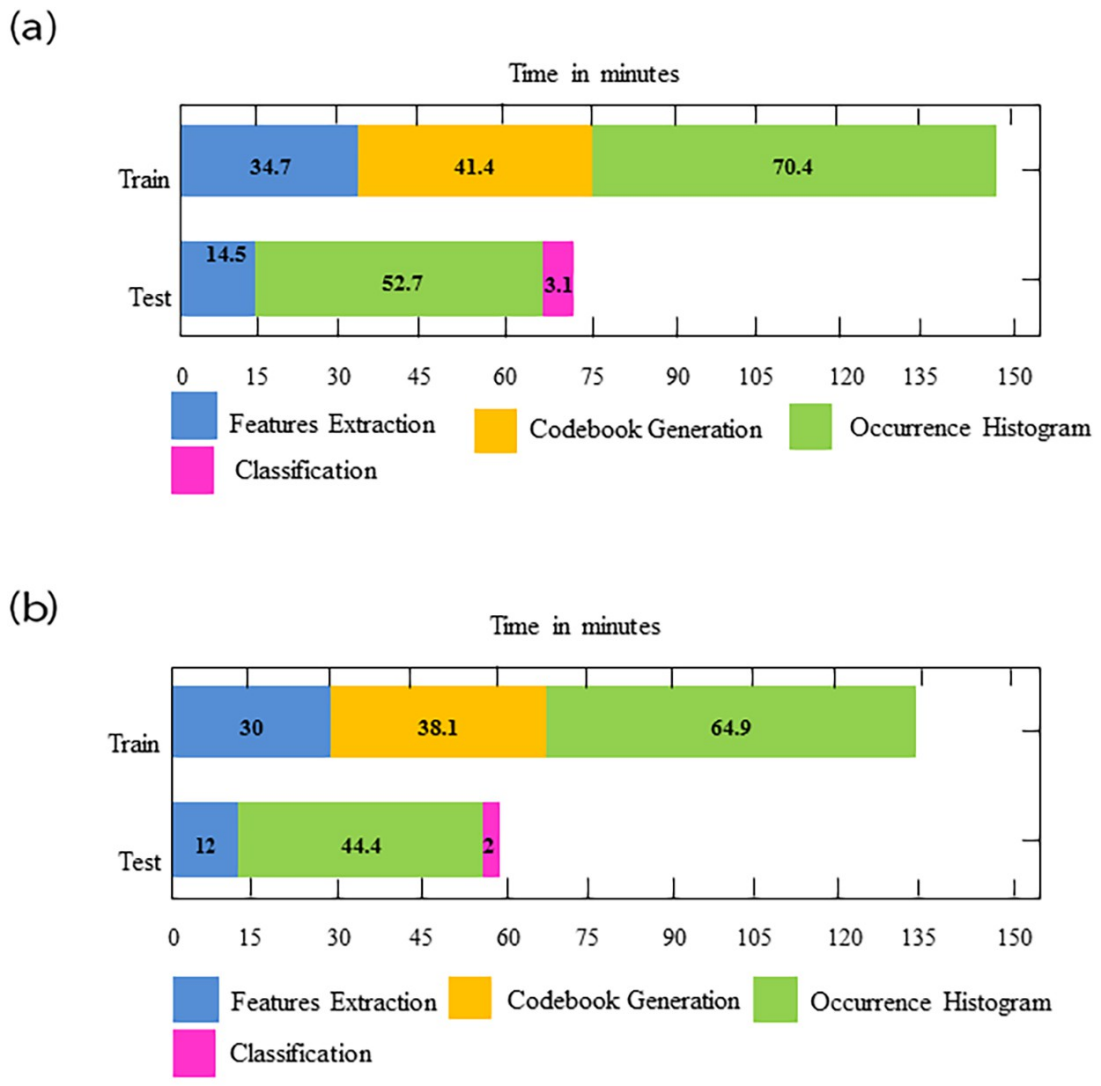
**(a)**. The runtime of different system steps on IAM dataset using CON^3^ Feature. **(b)**. The runtime of different system steps on IAM dataset using CPCA Feature.

**Fig 17:**
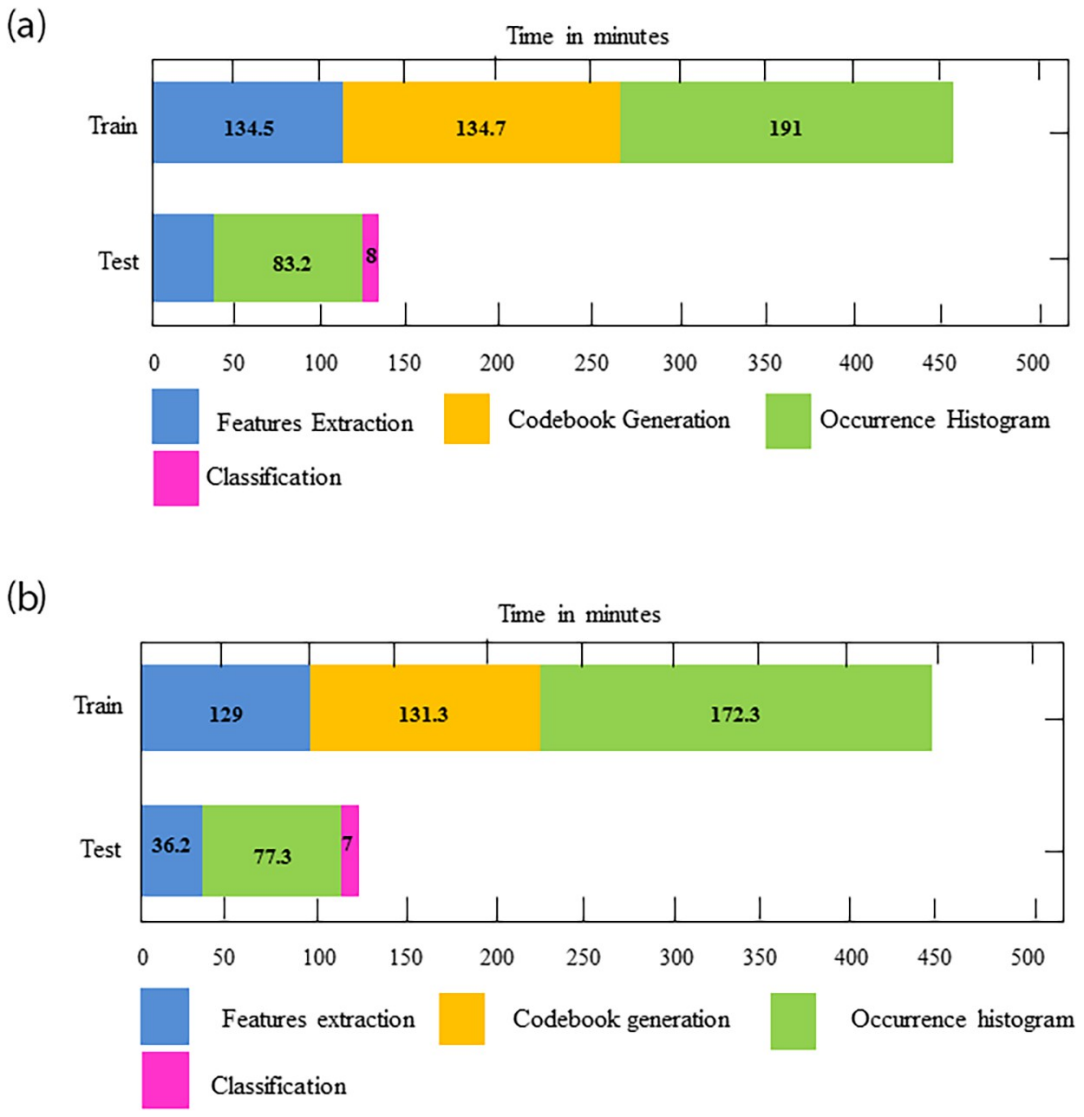
**(a)**. The runtime of different system steps on KHATT dataset using CON^3^ Feature. **(b)**. The runtime of different system steps on KHATT dataset using CPCA Feature.

Having the preprocessed data and setting up the system parameters to the optimal values enable us to measure the runtime cost of different steps of the system using both CPCA and CON^3^ features on IAM and KHATT datasets. As depicted in Figs 16 and 17, it is clear that the occurrence histograms calculation step takes much more time than other steps. That is because of the huge number of comparisons and distance calculations needed to build the histogram. This observation is consistent across the two datasets using both CPCA and CON^3^ features. Additionally, Figs 16 and 17 show that almost all processing steps of the system on KHATT dataset take quite more time than applying the same steps on IAM dataset. This observation is natural due to two reasons: (i) the size of the KHATT dataset in terms of the number of scribes is bigger than the size of IAM dataset. (ii) the forms of KHATT dataset are richer with text than the forms of IAM dataset, which in turn leads to producing a larger number of features. The processing time of the KHATT testing set was 120.5 and 131.4 minutes, i.e., each form almost takes 7.2 and 7.9 seconds using CPCA and CON^3^ features, respectively. It is worthy to mention that our implementation has not been optimized with respect to runtime, that is because the processing time is not considered a key performance indicator in offline type of systems [61]. Moreover, there is no need for real-time applications in the writer identification task (offline mode) [61]. It is difficult to compare the execution time with the benchmark methods due to the difference in the hardware and software configurations.

## 6 Conclusion and future work

This paper presented an effective method to extract two new features from the segmented contour fragments of offline handwritten images. Such features are intended to capture some structural properties of a handwritten contour, including concavity, convexity attributes, and curve angles. The extracted features were evaluated in the writer identification domain using the concept of a codebook.

Two public benchmarks, the Arabic KHATT and English IAM datasets are arranged to assess the identification rate of the proposed method and to compare it with the state-of-the-art methods. Empirical results showed that the combination of the CONtour point CONvexity/CONcavity (CON^3^) and the Contour point curve angle (CPCA) features scored the highest identification rate on both datasets, and the CON^3^ feature achieved better results than the CPCA feature on both datasets. Furthermore, to the best of our knowledge, the experimental results showed that the proposed method outperforms several state-of-the-art systems on the IAM dataset and provides very competitive results on the KHATT dataset.

This paper highlighted the impacts of common parameters, such as codebook size, available handwritten text size, and the number of writers involved in the experiment on the overall system identification rate. The major limitation of the proposed method lies in its dependency on some empirical internal parameters, such as segment size, gap, and angle intervals. These internal parameters need to be varied as functions of the underlying script. This limitation and the investigation of the big difference in the performance on the IAM and KHATT datasets will be addressed in the future. Finally, we intend to evaluate the proposed features in other issues of the text recognition domain.
